# Spatiotemporal asymmetries on brain energy landscape uncover system entrapment related to depression severity

**DOI:** 10.21203/rs.3.rs-7312306/v1

**Published:** 2025-08-19

**Authors:** B. Ülgen Kilic, Jenna Jubeir, Priti Balchandani, James W. Murrough, Laurel S. Morris, Yael Jacob

**Affiliations:** 1. Department of Psychiatry, Icahn School of Medicine at Mount Sinai, New York, NY, USA; 2. Center for Computational Psychiatry, Icahn School of Medicine at Mount Sinai, New York, NY, USA; 3. Nash Family Department of Neuroscience & Friedman Brain Institute, Icahn School of Medicine at Mount Sinai, New York, NY, USA; 4. BioMedical Engineering and Imaging Institute, Department of Radiology, Icahn School of Medicine at Mount Sinai, New York, NY, USA; 5. VISN 2 Mental Illness Research, Education and Clinical Center (MIRECC), James J. Peters VA Medical Center, Bronx, NY, USA; 6. Nuffield Department of Clinical Neurosciences, University of Oxford, UK; 7. Department of Experimental Psychology, University of Oxford, UK

## Abstract

We investigate the spatiotemporal organization of resting-state brain activity in individuals with and without major depressive disorder (MDD), identifying stable and recurring whole-brain functional co-activation patterns that serve as attractor-like configurations. A particularly prominent brain state, marked by suppressed default mode and frontoparietal networks and heightened salience system engagement, occurring more frequently and with shorter dwell times in MDD and correlating with greater anhedonia severity. Transition dynamics further reveal that MDD participants exhibit reduced transitions between visual-attentional and limbic-default mode systems, which is associated with higher overall depression symptoms, suggestive of affective and cognitive rigidity. By evaluating the dynamic properties in relation to white matter architecture, we find that MDD individuals preferentially transition along energetically costly trajectories, particularly from salience-reactive to introspective states, despite the presence of structurally facilitated alternatives, implicating inefficient structure-function coupling. Finally, characterizing the energetic asymmetries of entry and exit transitions uncovers the landscape on which the brain travels between source- and sink-like attractors, with MDD dynamics disproportionately becoming trapped between a local maximum and a deep basin. These results offer a mechanistic account of how depression may emerge from maladaptive state trajectories within an energetically imbalanced neural landscape.

## Introduction

Major depressive disorder (MDD) is a highly prevalent and clinically heterogenous psychiatric disorder^1^. Despite decades of neuroimaging research, current first-line treatments fail in over 30% of cases^2^ underscoring the need for reliable neural biomarkers to guide diagnosis, predict treatment response, and parse clinical heterogeneity^3^. Neuroimaging studies have identified group-level alterations in structural and functional connectivity, typically within the default mode (DMN), salience (SN) and frontoparietal (FPN) networks^4,5^. However, most of these findings are based on static connectivity measures, which overlook the inherently dynamic nature of brain function.

Resting-state fMRI studies have shown that spontaneous brain activity fluctuates over time, transitioning between distinct patterns of large-scale network co-activation^6,7^. Various methods have been proposed to quantify co-activation patterns (CAPs), including centroid-based clustering^8–11^, manifold learning^12,13^, hidden markov models (HMM)^14^ and topological data analysis (TDA)^15^, which attempt to capture brain dynamics by partitioning individual fMRI volumes based on a distance metric to define discrete and recurring brain states. Recent work applying CAP analysis to rs-fMRI data in individuals with MDD revealed increased time spent in DMN-dominant states, decreased time spent in FPN-related states, and greater transitions between DMN and FPN states, with these dynamics associated with symptom severity and specific depression symptomology^16–19^. While these findings provide strong support for the role of dynamic functional disruptions in MDD, they fail to address whether such disruptions are due to inefficient structure-function coupling^20^, or are independent of anatomical constraints.

Network Control Theory (NCT) offers a theoretical framework to study state trajectories by modeling transitions between brain states as a function of white matter connectivity^21–27^. Within this framework, each state transition is associated with a transition energy – total external input needed for the brain to transition between states. Recent work has shown that transitions between brain states that were empirically defined using fMRI occur more frequently when they require lower control energy, suggesting that white matter architecture may shape state trajectories^11^. Recent studies in healthy participants show^28,29^ that transition energies decrease after psilocybin, lysergic acid diethylamide (LSD) and dimethyltryptamine (DMT) infusions, yielding a less skewed, or flattened, energy landscapes, suggesting that the brain has more flexibility in altered states of consciousness, a key component in psychedelic therapies. However, this approach has not yet been applied to dynamic brain states in MDD to understand further the role of structural connectivity on disrupted functional dynamics.

Nevertheless, NCT is used to study how controllability of white matter topology is altered in MDD versus healthy controls, particularly within the DMN, FPN and attention networks, and related to memory and affective symptoms^30^. Reduced whole-brain modal controllability has been observed in MDD^31^, and baseline whole brain controllability has been used to predict electroconvulsive therapy (ECT) treatment response^32^. However, these studies primarily focus on global network metrics — such as average controllability — which quantify the theoretical ease of reaching any possible brain state, evaluating static architecture of connectomes rather than modeling dynamic transitions between empirically observed states. Understanding how structural scaffolding of the brain influence dynamics of brain states is crucial for bridging the gap between white matter topology and functional alterations observed in MDD.

**FIG1:**
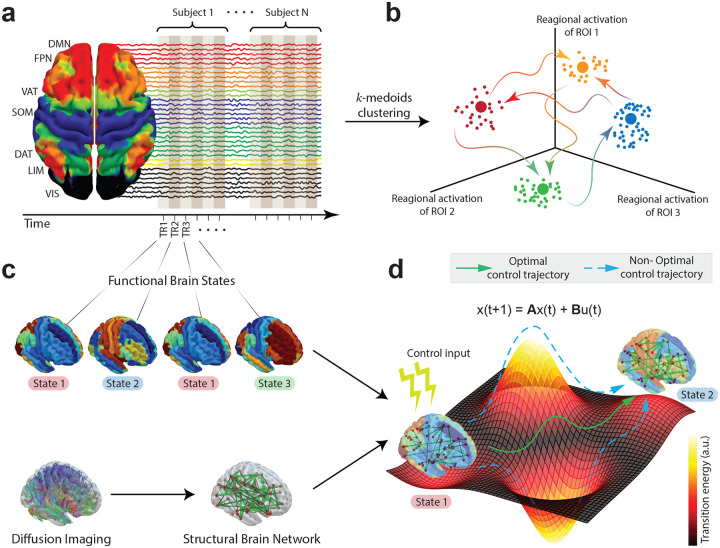
Brain revisits global and regional activation patterns through time. **a)** We collected 7T resting state fMRI from 38 healthy controls (HC) and individuals with 38 Major Depressive Disorder (MDD). By stacking all the rs-fMRI images along the temporal axis, we created a point cloud that we consider lying in an N dimensional ambient space, where N is the number of brain regions in the parcellation. **b)** We applied k-medoids algorithm to find cluster centers in our point cloud that correspond to representative fMRI volumes of spatiotemporal activations that are repeatedly revisited in time. **c)** We identified these representative points as brain states. In addition, we constructed subject-specific structural brain networks from a subset of the cohort (27 HC and 26 MDD) from which we also collected diffusion weighted imaging (DWI). **d)** We then adapted the network control theoretical framework to measure to what degree subject-specific structural connectomes allow participants to move between previously found brain states.

In this study, we combine coactivation pattern analysis of resting-state fMRI with network control theory applied to diffusion weighted imaging (DWI) to investigate how structure-function coupling shapes dynamic brain state transitions in MDD. By extracting ROI-wise time series and concatenating the signal across participants (Fig1a), we cluster fMRI volumes in a shared regional activation space that is native to subjects’ anatomical brain parcellations (Fig1b). Extracted cluster centers are characterized as temporally recurrent and spatially discrete coactivation patterns, i.e., brain states (Fig1c). We further model the specific transition energy required to move between empirically defined brain states via a linear, time-invariant diffusion equation informed by rs-fMRI. To do so, we employ network control theory to study white-matter connectivity of gray matter nodes, by extracting control signals, which, when theoretically applied to the respective control nodes, facilitate the transition between pairs of temporally recurrent fMRI activation maps (Fig1d). The extracted control signals can be summarized as a nodal control energy and a global transition energy characterizing the state transition in hand, and only dependent on the white matter connectivity, which then allows us to investigate whether transitions between fMRI-derived states are differentially constrained (or facilitated) by the structural connections in MDD, providing new insights into the relationship between structural connectivity, functional dynamics, and symptomatology.

## Results

### Canonical resting state networks described by spontaneous co-activation patterns

We applied the k-medoids algorithm^33^ for 50 iterations ranging k between 2 to 17 combined with correlation distance to the resting state fMRI scans of 38 healthy controls (HC) and 38 major depressive disorder (MDD) participants. We showed when K>5 not all brain states are well-represented in each participant across iterations (see Supplementary Figures 1e and 2c). Similarly, explained variance and mean medoid-silhouette coefficients^34^ started to level off around K=4,5 (Supplementary Figures 1b and 1d) and little additional variance was explained beyond K=4 ($r^{2}\;\text{gain}<0.01$ for K>4, Supplementary Figure 1c). Moreover, data points show a balanced distribution into 4 clusters (Supplementary Figure 1f) and show little to no spatial overlap with each other (Supplementary Figures 1g and 1h) when K=4. Importantly, the extracted cluster centers at K=4 show high reliability in a split-half resampling (Supplementary Figure 2a) and high recoverability in a populational-split where we cluster two populations separately (Supplementary Figure 2b). Additionally, we found that the BOLD signal showed significantly higher cluster quality and composition (measured by medoid silhouette coefficients and within cluster variance) than what would be expected from a random null model that generates signals with the same autocorrelation values^35^ (Supplementary Figures 2d, 2e and 2f). To further support our claim that K=4 is the optimal solution, we also extracted clusters for K=5 and matched two sets of clusters based on maximum Pearson correlation (Supplementary Figure 3a). This analysis showed that when K=5, cluster 2 and cluster 5 closely resembled to State 2 (r = 0.84, r = 0.58, respectively), suggesting redundancy. Lastly, providing evidence for the reproducibility of our results, we ran k-medoids clustering on an independent dataset with a cohort of healthy and MDD individuals for K=4 (Supplementary Figure 3b) and matched two sets of clusters using Pearson correlation. This showed that there was high degree of spatial overlap between the clusters found in both datasets (between State 1 and replicate cluster 1: r = 0.56, between State 2 and replicate cluster 2: r = 0.88, between State 3 and replicate cluster 3: r = 0.30, between State 4 and replicate cluster 4: r = 0.59), suggesting robustness.

To reduce the dimensionality of the regional activation space and to understand the 4 extracted brain states in simpler terms, we mapped above and below baseline components of the BOLD signal separately onto canonically defined resting-state networks^36^ by calculating cosine similarity between extracted spatio-temporal activation patterns and resting state networks in the native space of the subjects that the cluster centers belong to. Accordingly, State 1 showed a significant high amplitude activity on VIS and DAT networks combined with low amplitude activity spread across SOM, DMN, LIM networks. State 2 displayed a prominent high amplitude activity shared between FPN and DMN and low amplitude activity on VIS and DAT areas. State 3 was characterized by high amplitude activity shared between VAT, SOM and SUB areas with low amplitude activity around DMN and FPN network nodes. Lastly, State 4 showed a distributed high amplitude activity between LIM, DMN, VIS and low amplitude activity between VAT, FPN, SUB.

### Brain state dynamics are associated with depression severity

After identifying whole-brain co-activation patterns and their relationships with RSNs, we turned our attention to quantifying several properties of brain states throughout each subjects’ scan between two clinical populations. We used two metrics that have been established previously in the literature: ‘*dwell time*’, defined as the average duration of consecutive stays in each state per scan; and ‘*fractional occupancy*’, defined as the percentage of time frames identified as a given state^11^. We plotted the distributions of these two metrics for each clinical group and assessed the relationship between means of two groups using independent t-test. Moreover, we tested the hypothesis that if either of these quantities for states with significant group difference is correlated with self-reported MDD symptoms as measured by the Quick Inventory of Depressive Symptomatology (QIDS)^37^. To quantify the dimensionality of symptomatology of depression and rumination, we used the Mood and Anxiety Symptoms Questionnaire (MASQ) sub-scores: General Distress, Anhedonic Depression, and Anxious Arousal^38^, and the rumination tendency was assessed by the Ruminative Responses Scale (RRS) sub-scores: Depression-Related, Brooding, Reflection^39^.

We found that HCs dwell longer in State 3 compared to MDDs (independent t-test, $\mu_{HC-MDD}=1.262\;df=74,t=3.308,p=0.001$) as shown in Fig3a, whereas MDDs have higher fractional occupancy in the same state (independent t-test: $\mu_{HC-MDD}=-1.975\;df=74,t=-2.686,p=0.008$) as displayed in Fig3b. Furthermore, linear fits between MASQ-anhedonia scores and dwell times in State 3 for each clinical population suggests that increased dwell time in State 3 is inversely correlated with clinical anhedonia symptomatology for both HCs ($r^{2}=0.421,p=0.000038$) and MDDs ($r^{2}=0.261,p=0.042,$), whereas we did not find any relationship between dwell times in State 3 and QIDS depression scores (Fig3d) and other dimensions of the symptomatology (Supplementary Figure 6a). On the other hand, we found that fractional occupancies in State 3 were positively correlated with depression severity ($r^{2}=0.268,p=0.008$) and anhedonia symptomology ($r^{2}=0.284,p=0.008$) in MDDs, but not in HCs (Fig3e). No significant relationship was found between fractional occupancies in State 3 and general distress or anxious arousal symptomology or rumination in MDDs (Supplementary Figure 6b).

### Asymmetric state switching relates to anhedonia and rumination

The fact that people with MDD have higher fractional occupancy in State 3, but lower dwell times compared to healthy controls suggests that patients keep exiting and reentering to this state frequently. To test this hypothesis, we studied transition probabilities between each pair of states. We define state transition probability to a state $S_{i}$ at time point t+1, from the state $S_{j}$ at time point t, as the ratio of number of state switches from $S_{i}$ to $S_{j}$ to the total number of state switches from $S_{i}$ to other states after removing state autocorrelations (see Methods section for details). Moreover, we define exit and enter probabilities from $S_{i}$ to any other state and from any state $S_{i}$ as the average of all state probabilities from $S_{i}$ to other states and from other states to $S_{i}$, respectively. We additionally show that state transition probabilities that we study remains to be significant under random permutation testing, showing that transition probabilities are non-random (Supplementary Figure 4a).

In Fig 3c, we compare the exit probability from State 3 to any other state and enter probability from any state to State 3 between two groups and show that both of these events are more likely for the MDD group (independent t-test: for exit probability from State 3 $\mu_{HC-MDD}=-0.012,df=74,t=-2.537,p=0.013$ and for enter probability to State 3 $\mu_{HC-MDD}=-0.005,df=74,t=-3.169,p=0.002$). Furthermore, in Fig3e, we also compared these two quantities against QIDS and MASQ-AD scores and found that both exiting from and entering to State 3 are positively correlated with severity of MDD symptomatology (exit S3 vs. QIDS: $r^{2}=0.273,p=0.006$, exit S3 vs. MASQ-AD: $r^{2}=0.298,p=0.006$, enter S3 vs. QIDS: $r^{2}=0.186,p=0.011$, enter S3 vs. MASQ-AD: $r^{2}=0.210,p=0.008$). See the Supplementary Figure 6c and 6d for correlations with MASQ-AA and MASQ-GD scores. This result supports our previous finding that MDDs cannot dwell longer in State 3, as exit/enter probabilities of this state are significantly higher in MDDs, increasing MDD’s fractional occupancy in State 3, while preventing them to stay in this state for longer consecutive runs.

In Fig4a, a network diagram is presented in which the arrows and their colors represent state transitions and the difference in mean probabilities between groups (HC > MDD), respectively. For stability of results, we only focus on probabilities greater than 0.01. In Fig4b, we display distributions of each state transition probability in panel a) between groups and found that state transition probability from State 4 to State1 and State 1 to State 4 are lower in MDDs than HCs (independent t-test, from S4 to S1: $\mu_{HC-MDD}=0.021,df=74,t=2.590,p=0.011$, from S1 to S4: $\mu_{HC-MDD}=0.015,df=74,t=2.218,p=0.029$). On the other hand, transition probability from State 3 to State 2, and State 2 to State 3, are higher in MDDs than HCs (independent t-test, from S3 to S2: $\mu_{HC-MDD}=-0.020,df=74,t=-2.507,p=0.014$, from S2 to S3: $\mu_{HC-MDD}=-0.015\;df=74,t=-2.351,p=0.021$). These findings perhaps can be a by-product of increased state switching in and out of State 3.

Since state transition probability from State 4 to State 1 displayed the biggest difference between groups, we studied this transition in more detail by fitting linear models between transition probability from State 4 to State 1and several clinical anxiety and depression symptoms in Fig4c. We found that it is indeed the fact that as this transition probability increases, all the depression, anxiety and rumination scores decrease in MDD participants, suggesting that symptom severity is associated with lower State 4 to State 1 transitions (for QIDS: $r^{2}=0.285,t=-2.570$ and p=0.017, for RRS-Depression: $r^{2}=0.298,t=-2.706$ and p=0.017, for RRS-Brooding: $r^{2}=0.265,t=-2.351$ and p=0.024, for MASQ-anxiety: $r^{2}=0.287,t=-2.618$ and p=0.017, for MASQ-anhedonia: $r^{2}=0.313,t=-2.877$ and p=0.017 and for MASQ-general distress: $r^{2}=0.410,t=-3.836$ and p=0.003, Benjamini/Hochberg FDR corrected across number of comparisons). No significant relationship was found between adaptive rumination (RRS-Reflection) and this transition probability. We also show correlations between severity and transition probability from State 3 to State 2 in Supplementary Figure 6e.

### Structural connectivity modulates empirical state transitions

Next, we turned our attention to studying influence of white matter connectivity in state transitions. To do this, we leverage Network Control Theory which is a theoretical framework that computes control signals required to be exerted on to brain regions to move the brain from an initial neural activity configuration to a final configuration. NCT incorporates structural white matter connectivity obtained by diffusion weighted imaging between network nodes and extracts the signals under the assumption that BOLD activity propagates via a linear diffusion process in the brain. By integrating control signals along a time horizon that the state transition is maintained, we obtain a control energy in arbitrary units, a brain region specific statistic that describes the cumulative energy required to be exerted onto that network node (Fig5a and 5c). The sum of all control energies yields a total transition energy required to sustain a transition.

In a group mean comparison of the transition energies between HCs and MDDs for every pair of state transition, we did not find any significant results, yet most state transitions required higher transition energy on average for MDDs (Supplementary Figure 4b, 5a and 5e). However, control energy of Right-Putamen for healthy individuals was higher than MDDs as shown in Supplementary Figure 5c ($\mu_{HC-MDD}=0.501,t=4.04,df=51,p=0.015$ Benjamini/Hochberg FDR corrected).

By focusing on the transition from State 4 to State 1, we found that the more often this transition occurs in rs-fMRI of healthy individuals, the less costly it gets i.e., structural connectivity facilitates the transition from State 4 to State 1 when it more frequently occurs ($r^{2}=0.199,p=0.013$) (Fig 5b), yet this relationship was not found for MDDs. This suggests that white matter wiring of HCs promotes transitioning from State 4 to State 1 as we calculate lower transition energy, per unit increase in number of transitions made. On the other hand, we found that the transition from State 3 to State 2 was constrained by the structural connectivity for MDDs (Fig 5d), i.e., the more often this transition occur higher the energetic cost is (from State 3 to State 2: $r^{2}=0.152,p=0.040$), however, this relationships was not established for HCs. These relationships remained when we fit a combined line for both clinical groups with an interaction term between HCs and MDDs (from State 4 to State 1: $r^{2}=0.304,p=0.017$, from State 3 to State 2: $r^{2}=0.273,p=0.040$, Supplementary Figure 5d), which suggests that our results are driven by the group effect.

Additionally, comparing exit and enter energies for each state for the full cohort of participants with a DWI scan allows us to characterize the position of each empirically found brain state in the regional activation space relative to each other (Fig 5e). This conceptualization reveals topological gradients, trajectories of least resistance that naturally evolve i.e., uncontrolled, impulse response, where transitions along the gradient require less control energy than those against it^21,40^. For example, energy required for exiting State 1 is higher than entering State 1 due to anatomical constraints ($\mu_{\text{Exit}\;S1-\text{Enter}\;S1}=178.5,df=52,t=35.69,p=2.99\times 10^{-38}$, paired t-test), which would put State 1 on the bottom of a local minima because the brain would have to go up against the gradient of the energy landscape to exit State 1, which is energetically more costly, whereas state trajectories would have to follow along the gradient for entering State 1, which is more efficient in terms of transition energies. Similarly, we see a similar relationship between exit and enter energies of State 2 ($\mu_{\text{Exit}\;S2-\text{Enter}\;S2}=259.5,df=52,t=20.78,p=7.42\times 10^{-27}$, paired t-test), however, both energies are higher than the exit and enter energies of State 1, which puts State 2 on the bottom of a deeper local minima than State 1. On the other hand, exit energy required for State 3 is lower than entering State 3 ($\mu_{\text{Exit}\;S3-\text{Enter}\;S3}=-223.7,df=52,t=-26.43,p=8.10\times 10^{-32}$, paired t-test), which would position State 3 at a local maxima since one needs to exert more energy going against the gradient than going along the gradient. Similarly, exit energy of State 4 is lower than enter energy of State 4 ($\mu_{\text{Exit}\;S4-\text{Enter}\;S4}=-214.3,df=104,t=-34.02,p=3.30\times 10^{-37}$, paired t-test), however, both energies are lower than exit/enter energies of State 4, which would be characterized as a local maxima whose height is less than State 3 (Fig 5f).

## Discussion

In this study, we investigated the spatiotemporal dynamics of whole-brain activity during rest in individuals with and without major depressive disorder (MDD), identifying discrete functional states, evaluating their dynamical properties observed as temporal state sequences and their relationship with clinical symptoms of depression that differ between clinical groups. To characterize the latent organization of functional brain states, we represented each fMRI volume as a vector in a high-dimensional regional activation space. Using a robust centroid-based clustering framework, we partitioned the continuous BOLD time series into a finite set of recurring whole-brain activation patterns. The clusters, or “brain states,” reflect commonly visited configurations of activity and serve as functional units through which the brain traverses during rest. Though precise spatial activation coverages varied across individuals—reflecting inter-subject variability and inherent flexibility of brain function— the medoid-based approach robustly captured stable attractor-like configurations that recur across time and subjects. This abstraction allowed us to meaningfully compare brain dynamics across healthy controls and individuals with MDD.

A particularly salient finding was related to State 3, which was characterized by suppression of the default mode and frontoparietal control networks, alongside engagement of salience, subcortical, and somatomotor systems. This state occurred with higher frequency in MDD patients, yet maintained for shorter durations, indicating a pattern of increased recurrence, but reduced temporal stability. These temporal alterations were associated with greater anhedonia severity, suggesting a dysregulation in the balance between externally directed, goal-directed regulation (VAT) and internally directed, self-referential processing (DMN). Increased transition probabilities into and out of this state further highlight the dynamic instability and increased prominence of this state in depressive pathology. This finding is consistent with An et al^17^, who reported that below-baseline DMN states, marked by co-deactivated DMN and attention networks, occurred more frequently yet with shorter dwell times, suggesting altered attractor dynamics with deactivated DMN as a more accessible, yet less stable in MDD. However, this pattern contrasts with several prior CAP studies emphasizing increased dwell time in above baseline DMN states in MDD, which are typically associated and interpreted as heightened self-referential processing and ruminative thought patterns^16,41–43^. In this study, we found below baseline DMN states were closely linked to hedonic tone but not rumination.

Furthermore, patients with MDD exhibited altered dynamic transitions between functional brain states, showing reduced switching between State 4 and State 1—networks associated with visual-attentional integration and limbic-default mode modulation— which was also associated with greater depression and anxiety symptom severity in MDD group, while more frequently oscillating between State 3 and State 2. This compensatory pattern suggests that MDD individuals may become functionally ‘trapped’ in a loop characterized by hyperactivity in salience (ventral attention), somatomotor, and subcortical circuits (State 3), and alternating recruitment of frontoparietal and default mode networks (State 2). Prior work shows other examples of such entrapment pattern in subcortical regions following arousal^44^, as neural rigidity in autism^45,46^, or as biases and prejudices in one’s belief system^47,48^. Therefore, this finding may reflect impaired flexibility in navigating between perceptually grounded, externally oriented states and more introspective or affectively regulated states, possibly contributing to the deteriorating cognitive and affective flexibility seen in depression.

Overall, our findings in dynamic properties of temporal state sequences are consistent with hypotheses that depression may involve in aberrant attractor dynamics, wherein the brain exhibits reduced capacity to maintain stability within adaptive states and instead shifts more frequently between other reactive states^40,49^. Subsequently, a mechanistic interpretation of depressed brain dynamics can be that reactive and emotionally charged states (e.g., State 3) become dynamically unstable^50^ due to their intrinsically vulnerable position in the brain’s energy landscape^48,51,52^. This instability, exacerbated by genetic and environmental factors, increases the likelihood of transitions toward neighboring, more stable attractor basins—such as those corresponding to State 2. Over time this feedback loop forms a two-state attractor itself, which then self-reinforces over repetitive reimagination of the stressful, non-reward/punishment, life event in the cognitive/language systems, creating a metastable loop, narrowing the attractor repertoire and impairing neural flexibility^53^. Such oscillations between states are previously observed as limit cycles or heteroclinic orbits^54^ manifested as symptoms of mood related psychiatric conditions such as rumination and cognitive rigidity^55,56^. Therefore, State 4 and State 1 becomes decreasingly less available to transition in the state space due to this entrapment, which reflect aberrant control of responses to external emotional stimuli and impaired control of introspective, self-reflective thoughts, features commonly observed in depressive psychopathology.

Building on this theoretical framework, we sought to quantify how the brain’s structural wiring may shape these dynamic transitions. Importantly, we extended CAP analysis framework by integrating white matter tractography through network control theory (NCT) to model how structural connectivity constrains/facilitates transitions between extracted functional states^57^. Despite the presence of affective pathology, our findings revealed no statistically significant group differences in control energy. However, we observed a consistent trend toward higher mean transition energies in individuals with MDD (Supplementary Figure 5e).

In particular, NCT analysis combined with our clustering results revealed that individuals with MDD more frequently transitioned along energetically demanding paths—specifically, from State 3 (subcortical, salience network dominated) to State 2 (DMN/FPN dominated) —compared to more structurally facilitated transitions such as those from State 4 to State 1. It has been suggested that topology of the structural connectome is designed to facilitate efficient switching between brain states^11^, yet in some psychiatric disorders^27^, and in this study, MDDs’ preference for trajectories that require greater input to overcome structural constraints, despite the availability of lower-energy alternatives guided by the white matter scaffold was unexpected. This can perhaps be explained by the functional entrapment in the loop between State 3 and State 2, which become robust to bifurcations over time. As a result, although structurally facilitated transitions require lower control energy, brains with depression lose their ability to escape the basin of attraction of the two-state attractor and force the brains to follow structurally constrained trajectories. This finding my also explain some of the symptomatology seen in depression wherein MDD individuals rely on more costly transitions between reactive, salience-driven states (State 3) and cognitively burdened, introspective states (State 2), potentially reinforcing cycles of rumination (negative repetitive thinking). In contrast, healthy trajectories through structurally supported transitions—such as from emotionally integrated (State 4) to externally engaged states (State 1)—appear less accessible or less favored in depression, perhaps contributing to the observed difficulties in shifting attention, regulating affect, and maintaining adaptive cognitive control in MDD.

Lastly, by utilizing exit and enter energies of each state, we characterized energy landscape of 4 states respective to each other. Previous work studied energy landscapes within and between cognitive systems^26^, across clinical conditions^58^, and states of consciousness^28^. Different from these studies, our abstraction allowed us to characterize State 3 and State 4 as a local maxima from which exiting is easier i.e., a source-like attractor, whereas State 1 and State 2 as a local minima to which entering is easier, i.e., a sink-like attractor^40,49,51,54^. From this point of view, the tendency of individuals with MDD to repeatedly transition between State 3 and State 2 may reflect a dynamic imbalance, wherein the system oscillates between a fragile configuration (State 3) and a resilient deep basin (State 2), reinforcing maladaptive loops^47,48^. Conversely, the relatively underutilized transitions between State 4 and State 1 suggest existence of a cusp of energy barrier between the two trajectories. While it is easier for healthy brains to overcome this crux, likely due to the efficient functioning (e.g. usage of structurally facilitated trajectories), and relatively shallow positions of State 4 and State 1 in the energy landscape, depressed brains fail to jump over this barrier. Due to over exertion from utilizing structurally constrained trajectories, depressed brains remain stuck in the higher entropy hyperplane, consisting of the basins of attractions of State 3 and State 2, of the energy landscape. This conceptualization offers a mechanistic framework to understand how dysfunctions in a local attractor (State 3) may avalanche through and disrupt the global dynamics in an effort to compensate and adapt to the unexpected alterations.

It is important to note that, as defined by network control theory, transition energy quantifies the magnitude of external control input required to be injected into the brain under a fixed structural architecture. It does not, however, directly correspond to metabolic cost or neurophysiological energy consumption. This distinction positions transition energy as more analogous to a theoretical control input—such as the amplitude of deep brain stimulation^59,60^ or the pharmacodynamic strength of antidepressant interventions—than to intrinsic metabolic cost. From this perspective, characterizing transition energy landscape offer a unique, albeit abstract, framework to characterize brain dynamics, with potential translational relevance for understanding treatment dosage and responsiveness^61^. Additionally, we hypothesize that the absence of broader group effects may be attributable to the smaller sample size available for the diffusion weighted imaging dataset (n = 53), relative to the fMRI dataset (n = 76). Future work with larger diffusion imaging cohorts is warranted to clarify these preliminary observations and should aim to bridge this conceptual gap by directly correlating transition energy profiles with neuromodulatory treatments and clinically relevant biomarkers, including task-evoked fatigue and therapeutic response to targeted interventions.

## Methods

### Participants and Study Design

Participants with a rs-fMRI scan included 38 MDD patients (17 males, 21 females, mean age: 28.10 ± 6.68) and 38 HC (18 males, 20 females, mean age: 30.76 ± 8.74) age and gender-matched (p=0.14 and p=0.82, respectively). Among those, participants who also has DWI scan included 26 MDD patients (13 males, 13 females, mean age: 28.42 ± 6.59) and 27 HC (14 males, 13 females, mean age: 31.04 ± 8.00). The demographic and clinical characteristics are presented in Supplementary Table 1. All subjects were recruited at the Depression and Anxiety Center for Discovery and Treatment (DAC) at Icahn School of Medicine at Mount Sinai. All participants underwent the Structured Clinical Interview for DSM-V Axis Disorders (SCID-V) by a trained rater to determine any current or lifetime psychiatric disorder^62^. Subjects were excluded if they had an unstable medical illness (i.e., a significant, active medical condition that requires treatment), history of neurological disease, history of schizophrenia or other psychotic disorder, neurodevelopmental/ neurocognitive disorder, substance use disorder within the past 2 years, any contraindications to MRI, or positive urine toxicology on the day of the scan. HC subjects were free from any current or lifetime psychiatric disorder. Inclusion criteria for MDD subjects included having MDD as their primary presenting problem and being in a current major depressive episode. In all subjects, self-rated depressive symptom severity was measured by the Quick Inventory of Depressive Symptomatology, Self-Report (QIDS-SR)^37^ and the Mood and Anxiety Symptoms Questionnaire (MASQ)^38^ sub-scores (general distress, anhedonic depression, anxious arousal) were used to clinically quantify depressive symptoms. Rumination was measured by Rumination Response Scale sub-scores (depression related, brooding, reflection)^39^. All data were collected under Institutional Review Board (IRB) approved written informed consent and participants were compensated for their time.

### Data Acquisition

Structural, diffusion and functional MRI (fMRI) data were acquired using the ultra-high field 7T MRI scanner (Magnetom, Siemens) at the BioMedical Engineering and Imaging Institute, ISMMS, New York. T1-weighted anatomical scans were obtained using a dual-inversion magnetization prepared gradient echo (MP2RAGE) sequence with the parameters of repetition time (TR) =4500ms and time to echo (TE) =3.37ms. Resting state functional scans were collected during multi-band multi-echo fMRI with the following parameters: TR=2100ms, TE=14.0, 37.87, 61.74ms, 69 near axial slices, 1.5mm isotropic resolution, 10 min scan time. Multi-shell (1000, 2000) diffusion weighted imaging (DWI) data was acquired with a high-angular-resolved single shot spin echo EPI sequence with monopolar diffusion encoding with the following parameters: TR= 4000, TE=62 ms, 64 directions, 10 b-values from 0–2000 s/mm2, 1.05mm isotropic resolution, whole brain coverage, MB factor=2, in-plane GRAPPA acceleration R=3. Paired acquisitions with reversed phase encoding in the AP/PA direction was acquired at 9 min per scan for ~40 min DWI.

### MRI data preprocessing

Subject-specific T1-weighted anatomical images were processed using ConnectomeMapper3.1^63^ to obtain a nested parcellation of the brain with 85 cortical and subcortical brain regions in native space (scale 1, Lausanne parcellation). Multi-Echo Independent Components Analysis (ME-ICA) pipeline (https://bitbucket.org/prantikk/me-ica) is used for preprocessing, decomposition and denoising of the functional data. By acquiring multiple echoes, ME-ICA leverages the distinct echo time (TE) dependence of the blood oxygen level-dependent (BOLD) signal and various noise sources to enhance signal fidelity. The intensity of the BOLD signal will weaken as the TE increases because of T2* attenuation. Therefore, multi-echo acquisition enables characterization of the T2* decay curve by distinguishing neural activity-related signals from physiological or motion-related artifacts.

Diffusion data were preprocessed and denoised using MRtrix phase-reversed processing (https://mrtrix.readthedocs.io/en/latest/index.html). B1 field inhomogeneity correction is applied to the diffusion images followed by fiber orientation distributions (FODs) calculation from the diffusion data using spherical deconvolution^64^. The diffusion tensor was calculated using iteratively reweighted linear least squares estimator^65^. MRtrix software was used to obtain whole-brain probabilistic tractography^66^. Streamlines were thresholded with a 0.1 of FOD amplitude. The spherical deconvolution (SIFT2) algorithm was applied to all tracts to eliminate spurious streamlines that were unlikely to be physically accurate^67^.

### Time series extraction and connectome construction

To extract regional fMRI time series from subject-specific anatomical parcellations, each subject’s anatomical and functional data were aligned and resampled to a common space using AFNI^68^. First, a subject-specific brain mask, derived from the anatomical T1-weighted image was co-registered to the EPI space of the resting-state fMRI. The resulting transformation parameters were saved for subsequent use. Then, the Lausanne 2018 parcellation was aligned to the EPI space using the transformation parameters. The output parcellation was then resampled to match the resolution and grid of the EPI data. Finally, the transformed and resampled atlas was used as an ROI mask to extract mean time series from each region.

Similarly, DWI is coregistered to subject-specific structural T1-weighted anatomical images to enable anatomical parcellation in diffusion space. For each subject, the non-diffusion-weighted (b=0) volume from the DWI dataset was coregistered to the corresponding brain-extracted T1-weighted anatomical image using ANTs (Advanced Normalization Tools)^69^. A three-stage registration pipeline was employed, including rigid, affine, and symmetric normalization (SyN) transformations. The resulting transformation matrices were then used to warp the subject-specific parcellated anatomical atlas (L2018 Lausanne parcellation) into diffusion space with nearest-neighbor interpolation to preserve the discrete label values. Structural connectomes were generated for each subject, which mapped streamline endpoints to regions defined in the atlas. Whole brain tractograms and streamline weights from SIFT2 filtering were used to construct weighted connectivity matrices representing the structural connectome for each subject.

### Clustering of fMRI volumes

One simple, descriptive and unsupervised method of identifying clusters is called centroid-based clustering algorithms which is frequently used in data-oriented neuroscience literature^8,10,11,70^. The main principle of these methods is grouping a high-dimensional point cloud living in a regional activation space of $R^{N}$, in which each point consists of activation values of N regions of interests from a time point t of subject s, into global co-activation patterns based on distance between them. Importantly, distances between points are measured by the pairwise correlation between activation maps to quantify the degree of spatial similarity of brain activation maps i.e., coactivations, between different time points. Here, we normalize each subject-specific BOLD time series around mean 0 with standard deviation 1, and concatenate all resting state BOLD signals from 38 healthy controls and 38 people with MDD along the temporal axis in order to obtain an N×T matrix X where N is the number of ROIs in Lausanne2018 parcellation (85) and T is the total number of fMRI volumes across all participants (22315) (Supplementary Figure 1a). T consists of seventy-one ≈10min. scans (286 frames), three ≈15min. scans (420 frames), one ≈18min (519 frames). scan and one ≈8min. (230 frames) scan. We applied k-medoids^33^ algorithm to this matrix with correlation distance to cluster each time point in regional activation space living in $R^{N}$. This process yields cluster centers representing whole-brain spatiotemporal patterns of activity i.e., spatially distinct and temporally recurrent brain states. The choice of k-medoids algorithm assures cluster centers to be indeed one of the data points in our point cloud of fMRI volumes, as opposed to averaged values of all the data points in a cluster as in generally used k-means algorithm. This is important because average of points in a cluster may not necessarily represent BOLD activity which may affect the later analysis. Furthermore, k-medoids is more robust to outliers and more flexible in that it can work with any distance metric (correlation in our case), whereas k-means algorithm is sensitive to large variations in a dataset (in fact fMRI data is known to contain such outliers e.g. high-motion frames), and should only be used with distances that are consistent with arithmetic mean e.g. Euclidean distance.

To determine the number of clusters, present in the dataset, we ran k-medoids algorithm 50 times for k=2 to k=17. Since majority of our scans were ≈10minutes, with repetition time 2.1seconds, we determined that the theoretical limit for observing each state transition within a scan at least once is when $k=17\left(k^{2}>286=10\times 60÷2.1\right)$. To find the optimal number of clusters, K, we calculated explained variance, variance gain, medoid-silhouette coefficients and the percentage of subjects in which at least one state is absent across 50 repetitions as a function of K Supplementary Figures 1b–e. We first heuristically decided that the elbow is around K=4,5, and gain in variance started to taper below 0.01 for unit increase in K after K=4. To assess the cluster quality, we looked at mean medoid-silhouette scores and individual medoid-silhouette plots (Supplementary Figure 1f) which reveal that clusters are not as balanced as K=4 when K>4 in terms of composition and distribution of fMRI volumes in each cluster. We additionally wanted to make sure all clusters to be represented in all our cohort (Supplementary Figures 1e and 2c). Finally, by computing pairwise correlation between each pair of states, we found that extracted cluster centers start spatially overlapping with each other for K>4 (Supplementary Figure 1h), concluding K=4 was the optimal number of clusters in this dataset. Lastly, for the rest of our analysis, we proceed with the clusters found in the iteration with maximum inertia at K=4.

To verify the robustness and stability of our results, we conducted three additional tests: a random split-half validation of cluster centroids, a populational split of cluster centroids and comparison of cluster quality metrics with an appropriate null model. For the former, we split our point cloud matrix X in two equal halves after 500 random permutations and obtained cluster centers in each half as described above. Then, we mapped cluster centers in both halves onto each other based on maximum cross-correlation values which we plotted in Supplementary Figure 2a and found that most pairs of clusters centers are showing very high degree of similarity (r>0.95). Additionally, by running k-medoids algorithm only on HCs and only on MDDs, we were able to recover extracted cluster centers in the main text with almost 100% accuracy (Supplementary Figure 2b). This analysis showed extracted cluster centers are robust to outliers and consistent across different subsamples of our dataset. Next, to test the hypothesis that our cluster are in fact more likely to form than random, we sampled independent phase randomized (PR) null time series^35^ based on our fMRI scans of cohort of 76 subjects. We ran k-medoids algorithm as described above on this null dataset and then compared medoid-silhouette coefficients and found that $\mu_{\text{real}}-\mu_{\text{null}}=0.117$ with $p<10^{-200}$ (Supplementary Figure 2e). We also compared intra-cluster variance for each cluster between real data and the null model and found that within cluster variances for each cluster in our dataset were significantly higher than the null distribution ($\mu_{\text{real}}^{0}-\mu_{\text{null}}^{0}=0.07,\mu_{\text{real}}^{1}-\mu_{\text{null}}^{1}=0.05,\mu_{\text{real}}^{2}-\mu_{\text{null}}^{2}=0.05$ and $\mu_{\text{real}}^{3}-\mu_{\text{null}}^{3}=0.05$, for clusters 0 through 3 all $p<10^{-200}$, Supplementary Figure 2f). These analyses suggest that BOLD data exhibit non-trivial and non-random activation space and suggests that the quality and composition of our clusters are more stable than random.

### Characterization of temporal state dynamics

**FIG2:**
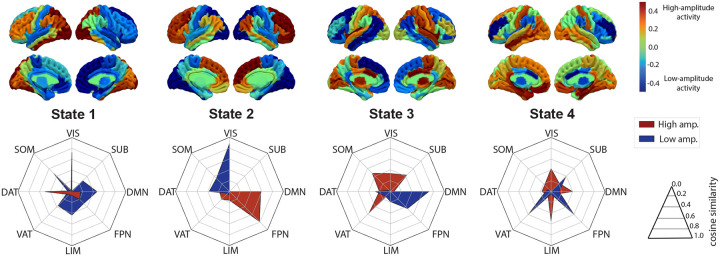
Temporally recurrent and spatially discrete brain states. We show left/right hemispheres of sagittal/lateral views of the 4 brain states (averaged across all points in each cluster) identified by k-medoids algorithm (top). Below each state, we show a radar plot in which high amplitude (positive z-score, red) and low amplitude (negative z-score, blue) BOLD signals are mapped onto canonical resting-state networks (RSNs) using cosine similarity. Each state is described by a configuration of the canonical RSNs that they map onto. VIS-Visual network, SOM-Somatomotor network, DAT-Dorsal attention network, VAT-Ventral attention network, LIM-Limbic network, FPN-Frontoparietal network, DMN-Default mode network and SUB-Subcortical network.

**FIG3:**
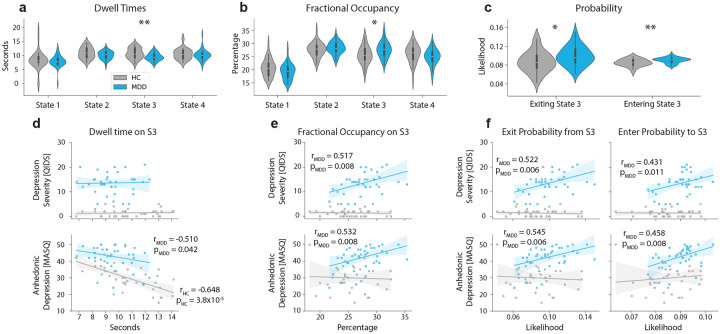
Dynamic properties of State 3 are predictors of clinical depression and anhedonia. We compare **a)** dwell times in seconds and **b)** fractional occupancies in percentages of 4 brain states that we established between healthy controls (HC) and major depressive disorder (MDD) participants. **p<0.01,*p<0.05, independent t-test Bonferroni-corrected across K=4 states separately for each quantity. HCs, on average, dwell longer on State 3 than MDDs, whereas MDD individuals spend longer overall duration in this state compared to HCs. Ordinary least squares regression with age, gender and medication use added as a covariate, is fit between **d)** dwell times on State 3 and clinical depression (QIDS, top) and anhedonia scores (MASQ-AD, bottom), and **e)** fractional occupancy of State 3 and QIDS (top) MASQ-AD scores (bottom) for each subpopulation separately. We also show probabilities of exiting from and entering to State 3 in **c)** and linear fits between these probabilities and QIDS (top) and MASQ-AD scores (bottom) in **f)**. p-values in panels **d)**, **e)**, and **f)** are controlled for multiple comparisons across clinical scores. QIDS- Quick Inventory of Depressive Symptomatology, MASQ-AD- Mood and Anxiety Symptoms Questionnaire Anhedonic Depression sub-score.

**FIG4:**
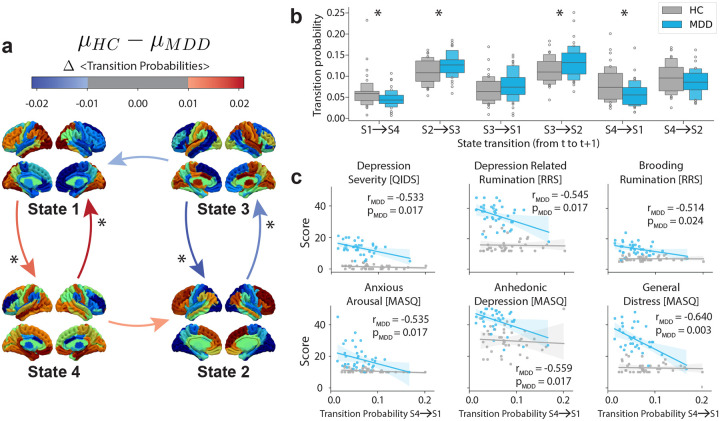
Transition probabilities between each pair of states. **a)** State transition diagram, in which arrows between 4 states represent differences in average transition probabilities between groups (HC > MDD). **b)** The distributions of each transition probability (shown in a) between HCs and MDDs as a box plot in which every small box above the next level contains 50% of the remaining data points. We found that transition probabilities from State 1 to State 4, and vice versa, were significantly more frequent among HC, whereas transitions from State 2 to State 3, and vice versa, were significantly more frequent among MDD. p*<0.05 independent t-test Benjamini/Hochberg FDR corrected. **c)** Associations between transition probabilities from State 4 to State 1 and clinical anxiety and depression scores (QIDS, RRS-Depression-Related, RRS-Brooding, MASQ-anxious arousal, MASQ-anhedonic depression and MASQ-general distress) of HCs and MDDs while controlling for age, sex and medication use, and controlling for number of comparisons performed. Note that this particular transition probability is higher in HCs than MDDs, and higher transition probability associates with lower depression symptoms for MDDs.

**FIG5:**
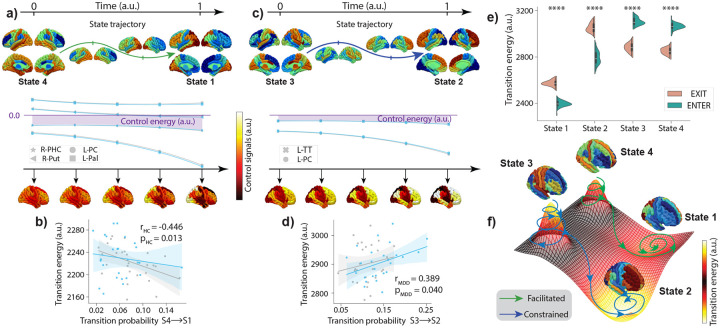
Transition energies characterize relative energy landscape between empirical states. We apply control theoretic framework to structural connectomes for studying state transitions. Transition from State 4 to State 1 **a)** and from State 3 to State 2 **c)** (between cluster centers) is depicted with obtained functional state trajectories (top) and extracted structural control signals (bottom) for a select ROIs which show uncorrected significant group difference. Control energy corresponds to the area under the control signal curve (in purple). Linear fits between transition energy and transition probability of transition from State 4 to State 1 **b)**, from State 3 to State 2 **d)** are shown. **e)** Violins compare exit and enter energies between all 4 states, p****<10-25 paired t-test Bonferroni corrected, which characterizes the relative positions of each state in the energy landscape in **f)**. (L-Pal: Left-pallidum, R-Put: Right-putamen, L-PC: Left paracentral lobule, R-PHC: Right parahippocampal gyrus, L-TT: left-transversetemporal gyrus).

After identifying 4 spatially non-overlapping and non-correlated cluster centers, we averaged all the time points that belong to each cluster to generate 4 brain states in Fig 2. Next, to understand the cortical coverage of each state in terms of canonically defined resting-state networks, we used Yeo7^36^ partition by co-registering this predefined atlas into the native spaces of the 4 subjects that contain the cluster centers. Since our parcellations have additional subcortical coverage that Yeo7 networks do not, we mapped all the subcortical areas as an additional resting state network ‘Subcortical’ in subject specific native spaces. Next, since each cluster center is one TR worth of BOLD signal centered around 0 with standard deviation 1, we mapped positive (above baseline) and negative (below baseline) components of spatiotemporal activation patterns on to canonical resting state networks based on the cosine similarity between them. See Supplementary Table 2 for a list of ROIs, components of activity on each ROI for each State, and mapping of each ROI to a cognitive system.

Initially, two statistics describing the distribution of 4 states across subjects are computed: 1) state dwell time in seconds defined as the consecutive appearance of a given state on average per subject, 2) state fractional occupancy given by percentage of a given state throughout a scan per subject. We additionally studied transition probabilities between state i at time t and state j at time t+1 after removing self-transitions. Here, we did not include state transitions between different subjects. We also showed that the group differences we observed are non-random via permutation testing in Supplementary Figure 4.

### Network Control Theory and Dynamics on Networks

Network Control Theory is a powerful tool that operates on dynamical systems principles. The main idea of NCT is similar to simulating functional dynamics on structural connectomes, which mimics neuronal activity in the brain along a spatio-temporal trajectory starting at point $x_{0}$ and terminating at $x_{f}$ due to an external control input u(t). However, NCT achieves this task without explicitly simulating this trajectory. Instead, it tries to solve a simple, linear, time-invariant diffusion equation defining the functional dynamics

$$\frac{d}{dt}x(t)=Ax(t)+Bu(t)$$

for u(t) for given $x_{0}$ and $x_{f}$, initial and terminal points in the regional activation space, respectively. Here, the matrix A is a symmetric, weighted, subject-specific N×N structural connectivity matrix obtained by DWI in which $A_{i,j}$ is the white matter fiber strength between brain region i and j. To make our system well-defined, we set $A_{i,j}=0$ whenever i=j. The matrix B is an N×m matrix that constraints the control signals on the subset of nodes. Here, we take m=N and B as the identity matrix, i.e., all nodes of the network have equal contribution to the controlled dynamics, uniform full control set. We set up other control parameters according to *optimal control*^21^ i.e., ρ=1 and trajectory constraints as N×N identity matrix) and calculated state trajectories and transition energies between non-binary states along a continuous time horizon T=1. Lastly, we define exit and enter energies similarly to exit/enter probabilities (e.g. exit energy from State 1 is the mean of transition energies from State 1 to State 2, State 1 to State 3 and State 1 to State 4).

### Statistical Inference

In general, we compared the means of calculated statistics throughout the text between two clinical groups using independent t-test. We also utilized random permutation testing when it is not possible to establish a baseline for extracted quantities. When reporting p-values, we either used Bonferroni-correction or FDR-correction across number of comparisons made.

Lastly, we fit ordinary least square regression to assess relationships between calculated statistics and clinical depression and anxiety symptoms using the following model:

$$Q=\beta_{0}+\beta_{c_{x}}c_{x}+\beta_{s}s+\beta_{a}a+\beta_{m}m+\epsilon$$

where $c_{x}$ is a given clinical score, s is sex, a is age, m is medication use and ϵ is an error term and Q is a measure of calculated statistics such as dwell time, fractional occupancy or transition probability.

## Supplementary Material

1

## Data Availability

The authors declare that all data supporting the findings of this study are available within the paper.
