# Supplementary material for: Spatiotemporal asymmetries on brain energy landscape uncover system entrapment related to depression severity
Source: Res Sq. 2025 Aug 19:rs.3.rs-7312306. Preprint. [Version 1] doi: 10.21203/rs.3.rs-7312306/v1 (PMC12393491; doi:10.21203/rs.3.rs-7312306/v1)
Supplement: 1 [file NIHPPRS7312306V1-supplement-1.pdf]

Supplementary Materials: Spatiotemporal asymmetries of brain energy landscape uncover system entrapment related to depression severity

B. Ülgen Kilic*[1,2], Jenna Jubeir[1,3], Priti Balchandani[3,4], James W. Murrough[1,3,5], Laurel S. Morris[1,6,7], Yael Jacob[1,3,4]

1. Department of Psychiatry, Icahn School of Medicine at Mount Sinai, New York, NY, USA
2. Center for Computational Psychiatry, Icahn School of Medicine at Mount Sinai, New York, NY, USA
3. Nash Family Department of Neuroscience & Friedman Brain Institute, Icahn School of Medicine at Mount Sinai, New York, NY, USA
4. BioMedical Engineering and Imaging Institute, Department of Radiology, Icahn School of Medicine at Mount Sinai, New York, NY, USA
5. VISN 2 Mental Illness Research, Education and Clinical Center (MIRECC), James J. Peters VA Medical Center, Bronx, NY, USA
6. Nuffield Department of Clinical Neurosciences, University of Oxford, UK
7. Department of Experimental Psychology, University of Oxford, UK

Determining optimal number of brain states

We ran 50 initial iterations of k-medoids algorithm[1] with correlation distance via python scikit-learn-extra implementation[2] on our point cloud $\mathbf{X}$ (Supplementary figure 1a) while ranging K from 2 to 17 and calculated explained variance (Supplementary figure 1b), variance gain (Supplementary figure 1c) and medoid-silhouette coefficients (Supplementary figure 1d)[3]. Explained variance is defined as the between-cluster variance divided by the total variance (within-cluster variance plus between cluster variance)[4,5], while gain in variance is calculated as the discrete derivative of explained variance. We defined percentage of absent states as the ratio of number of subjects in which at least one brain state is not represented in their fMRI time series to the total number of cluster representations across iterations. We calculated percentage of absent states in the full cohort (Supplementary figure 1e) and in each clinical group separately (Supplementary figure 2c) for each K between 2 and 17, and found that there is at least one subject in an iteration that is missing at least one state when $K > 4$. Lastly, we plotted spatial correlations between each pair of states for every K (Supplementary figure 1h) to understand at which K value extracted clusters started to spatially overlap with each other. This analysis showed for $K > 4$, at least 1 pair of clusters were spatially overlapping. Collectively, these results support the choice of $K = 4$ for the analysis in the main text.

Cluster stability and robustness to outliers

We performed a random split-half validation[6] of cluster centroids as follows. We split the point cloud $\mathbf{X}$ shown in (Supplementary figure 1a) in half 500 times and extracted cluster centers in each half across iterations using k-medoids algorithm with correlation distance. Then we mapped cluster centers based on maximum cross correlation between each half. We plot these correlation values in (Supplementary figure 2a). Additionally, we performed a population split $\mathbf{X_{HC}}$ and $\mathbf{X_{MDD}}$ such that $\mathbf{X_{HC}} + \mathbf{X_{MDD}} = \mathbf{X}$ where $+$ operation denotes concatenation along the horizontal axis. Then, we used k-medoids algorithm separately on each clinical group to test the hypothesis that if the cluster centers are recoverable independent from each subpopulation. Then, we again matched the cluster centers based on maximum spatial correlation similarity between the clinical groups (HC and MDD) and the full cohort (HC + MDD) and arranged matching clusters so that the cluster indices found in the full cohort matches with the indices found in subpopulations (Supplementary figure 2b). Observe that cluster 3 found in $\mathbf{X_{HC}}$ has the highest correlation value $(r = 0.999)$ with the original cluster 3 since this cluster was a time point originally belonging to an HC. Similarly, clusters 1, 2 and 4 found in $\mathbf{X_{MDD}}$ has the highest correlation value with the original clusters 1, 2 and 4 ($r = 0.824$, $r = 0.996$ and $r = 0.998$) with the original clusters 1,2 and 4 belonging to MDD patients. This finding suggests that cluster centers are not randomly found and can be recoverable across independent clustering runs.

To test the hypothesis that the extracted clusters are nonrandom, we simulated 50 independent phase randomized (PR) null time series series[7] using our dataset. This null model is shown to generate rsfMRI-like signals that are linear, weak-sense stationery and Gaussian, breaking any inter-regional covariance apart while keeping intra-regional autocorrelation intact, hence, is a good candidate for representing dynamic functional connectivity. We ran k-medoids algorithm on each sle separately for $K = 4$ and picked the sle with maximum inertia to compare medoid-silhouette coefficients and intra-cluster variance between the null sle and the original dataset. Assuming that every point is assigned to a cluster, medoid-silhouette coefficient of a point i can be simplified to $1 - \frac{a_i}{b_i}$ where a_i and b_i is the distances (in correlation) between the point i and the nearest and the second nearest cluster centers, respectively. If $b_i = 0$, then medoid-silhouette coefficient is 1, and mean medoid-silhouette coefficient is defined as the average medoid-silhouette coefficient across all data points. Higher values of medoid-silhouette coefficient is interpreted as the data points being well-assigned to their clusters, versus lower values indicate weak cluster assignments. By comparing silhouette plots in Supplementary figure 1f and 2d, one can observe that the silhouette profile of the null data show very weak silhouette scores across all data points in each cluster, especially for cluster 1. Additionally, these silhouette coefficients are plotted against each other for visual comparison in Supplementary figure 2e and one can see that the mean silhouette score of the real data is significantly higher than random ($p < 10^{-200}$ with independent t-test). Additionally, within-cluster variance values for each cluster between the real data and the IPR sle show that the randomly generated data has very little obvious structure as the average intra-cluster values are all significantly lower than the real data for each cluster (all $p < 10^{-50}$ with independent t-test Bonferroni corrected across 4 states) Supplementary figure 2e.

Influence of number of clusters

The number of clusters in centroid-based clustering algorithms is the only free parameter that impacts the analysis since the following analysis depends on the extracted clusters. As justified above, the choice of $K = 4$ was the optimal solution for this problem, yet we analyzed cluster centroids for $K = 5$ also in this section. In Supplementary Figure 3a, we project average activity

of each of 5 clusters on to anatomical brain surface with a corresponding radar plot mapping the activity onto canonically defined resting state networks described in the Methods section of the main text. Below each brain, we show a heatmap showing degree of spatial similarity with each of the 4 states studied in the main text. Our analysis shows that States 1 through 4 are mostly recovered when $K = 5$ and an additional Cluster 5 is found. The extra state shows a prominent high-amplitude activity on DMN and low-amplitude activity distributed between VIS, SOM, DAT and VAT networks, which shows similarity with State 2 as shown by the heatmap. Given that both Cluster 2 and Cluster 5 was resembling State 2, our result reinforces our initial choice that $K = 4$ was indeed the optimal number of clusters since we would want our states to be as non-overlapping as possible.

Brain state reproducibility in an independent dataset

To provide evidence for the reproducibility and generalizability of our findings, we applied the same k-medoids clustering procedure to an independent validation dataset comprising both healthy controls and individuals with major depressive disorder (MDD), using the same number of clusters (K=4; Supplementary Figure 3b). All information about this dataset can be found in Jacob et.al.[8]. To assess the correspondence between the brain states identified in the discovery and replication datasets, we calculated spatial similarity between the resulting cluster centroids using Pearson correlation. This analysis revealed a substantial degree of spatial overlap between matched clusters across the two datasets, indicating that similar co-activation patterns reliably emerged. Specifically, the correlations were r = 0.56 for State 1 and replicate cluster 1, r = 0.88 for State 2 and replicate cluster 2, r = 0.30 for State 3 and replicate cluster 3, and r = 0.59 for State 4 and replicate cluster 4. These results suggest that at least a subset of the identified brain states—particularly States 1, 2, and 4—represent robust, recurring patterns of brain-wide co-activation that generalize across cohorts.

Comparing transition probabilities with null models

To show non-randomness of state transition probabilities, we generated $100{,}000$ permutations of each sequence of self-transition-removed state labels for each subject and computed average state transition matrices of shape $100{,}000x4x4$ for each group. We obtained a p-value for every state transition by measuring how frequently the observed difference was larger/smaller than in the null distribution. We followed a similar procedure for computing exit, enter and persistence probabilities of the null distribution as we did for the observed values for these quantities, and show that the group differences of the probabilities we studied in the main text are more likely to occur than by chance.

In Supplementary Figure 4a), we show difference in observed mean transition probabilities between groups for all transitions. As mentioned in the main text (Fig 4b), transition probability from State 4 to State1 and State 1 to State 4 are higher in HCs than MDDs, whereas transition probability from State 3 to State 2 and State 2 to State 3 are lower in HCs than MDDs. Our permutation testing shows that these differences do not occur by chance (from S4 to S1: $p = 0.0007$, from S1 to S4: $p = 0.008$, from S3 to S2: $p = 0.040$ and from S4 to S3: $p = 0.050$). Moreover, in the main text, we mentioned that exit and enter probabilities of State 3 are higher in MDDs, and, both events are more likely to occur than by chance as shown by permutation testing from (Exit from S3: $p = 0.0006$, Enter to S3: $p = 0.008$). Additionally, focusing on exit and enter probabilities of State 1 in the observed data show that HCs enter to and exit from this state more often than MDDs (Exit S1: $\mu_{HC-MDD} = 0.008$, $df = 74$, $t = 2.093$, $p = 0.039$ and Enter S1: $\mu_{HC-MDD} = 0.004$, $df = 74$, $t = 2.059$, $p = 0.042$ independent t-test), and permutation testing show that these events are not occurring by chance (Exit from S1: $p = 0.004$, Enter to

S1: $p = 0.042$). We also found that Exit probability from State 4 is more likely to occur than by chance, although there is no group difference in the observed data (Exit S4: $p = 0.004$). Finally, we observe that persistence probabilities in State 3 and State 4 are more likely to occur than by chance, yet there wasn't any group difference in the observed data.

Network control theory

We use network control theory to assess influence of structure on transitions between empirically extracted brain states[9]. To characterize brain dynamics, we implemented a linear, continuous-time, and time-invariant model constrained by the brain's structural connectivity derived from diffusion-weighted imaging. The structural network is represented as a graph $G = (V, E)$ where V denotes the set of brain regions (nodes) and E the set of connections (edges) between them. The strength of each connection is given by a weight $A_{i,j}$ defined for each edge $(i, j) \in E$. These weights, based on streamline counts, form the entries of the weighted adjacency matrix A with no self-loops, i.e., if $i = j$, $A_{i,j} = 0$.

We define a time-dependent vector $x(t) \in \boldsymbol{R}^n$ representing the evolving activity levels across $n = 85$ brain regions, where each element x_i^t corresponds to the activity in region i at time t. The dynamics of the system are modeled by the following equation:

$$\frac{d}{dt}\mathbf{x}(t) = \mathbf{Ax}(t) + \mathbf{Bu}(t)$$

Where left hand-side is the time derivative of $x(t)$, A is the structural connectivity matrix, $u(t)$ is the external control input to be computed, and B is the input matrix determining how control influences each region. In our analysis, B was set to the identity matrix I_n allowing each region to be independently controlled with a uniform weight.

To ensure the system is marginally stable—i.e., its dynamics neither diverge nor decay uncontrollably—we normalized the connectivity matrix A. Specifically, we divided A by its largest eigenvalue λ_{max} plus a normalization constant c and then subtracted the identity matrix:

$$A_{normalized} = \frac{A}{\lambda_{max} + c} - I_n$$

We set $c = 1$ by default, which ensures that all modes of the system decay and thus that activity goes to zero over time, yielding marginally stable dynamics suitable for evaluating control energy. We additionally normalized both the initial and final state vectors, $x_0(t)$ and $x_f(t)$ prior to computing control energy. Specifically, we converted the state vectors to floating-point representations and applied Euclidean (L2) normalization:

$$x_{normalized} = \frac{x}{||x||_2}$$

This normalization constrains the state vectors to lie on the unit hypersphere, ensuring that control energy reflects differences in configuration rather than overall magnitude. To compute optimal control energy, we also set trajectory constraints as the identity matrix I_n and allow equal cost over the magnitude of the control signals and the state trajectory by setting $\rho = 1$.

To compute the optimal control energy required to drive the system from an initial activity pattern $x_0(t)$ (when $t = 0$) to a final activity pattern $x_f(t)$ (when $t = T$), we compute an invertible controllability Gramian W for controlling the network A from the set of network nodes:

$$W = \int_0^T e^{\boldsymbol{A}(T-\tau)} \boldsymbol{B}\boldsymbol{B}^{\boldsymbol{T}} e^{\boldsymbol{A}^{\boldsymbol{T}}(T-\tau)} d\tau$$

where $T = 1$ is the time horizon in the continuous model, which specifies the time over which input to the system is allowed. After computing the controllability Gramian, we can solve for the optimal control energy E_o by computing the quadratic product between the inverted controllability Gramian and the difference between x_0 and x_f:

$$E_o = \left(e^{AT} x_0 - x_f\right)^{\boldsymbol{T}} W^{-1}(e^{AT} x_0 - x_f)$$

We computed transition energies between all possible state transitions as described above. Supplementary Figure 4b shows these transition energies as a $4x4$ matrix. We did not find any group difference between HCs and MDDs (Supplementary Figure 5a), yet we observed that MDDs require higher transition energy than HCs for almost all transitions except the transition from State 2 to State 3 and State 4 to State 3. We summarize this result as MDDs require higher mean transition energy than HCs ($t = -1.41$, $p = 0.16, df = 51$) (Supplementary Figure 5e). We additionally computed exit and enter energies defined as the sum of columns and rows of the transition energy matrix, respectively, as well as persistence energies which quantify the energy required to maintain $x_0(t)$, in the case where $x_0 = x_f$ (Supplementary Figure 4b). Similarly, we did not find any group difference between exit and enter energies (Supplementary Figure 5b).

Our results mainly focused on two state transitions that show the largest group difference in transition probabilities. First one is the transition from State 4, $x_0(t)$, to State 1, $x_f(t)$, (Fig 5a in the main text) which showed biggest difference in transition probabilities skewed towards HCs, and the second one is the transition from State 3, $x_0(t)$, to State 2, $x_f(t)$, (Fig 5c in the main text) which showed the biggest difference in transition probabilities skewed towards MDDs. Neither of these transitions showed significant group difference in transition energies as mentioned above. Yet, our results indicate that transition energies and transition probabilities are inversely correlated for HCs for the former state transition, whereas they are positively correlated for the latter state transition. In Supplementary Figure 5d, we additionally show linear fits between both of these correlations independent of the clinical group, and show that transition energies and transition probabilities are positively correlated for transition from State 4 to State 1 ($r^2 = 0.093$, $p = 0.017$) , and negatively correlated for the transition from State 3 to State 2 ($r^2 = 0.075$, $p = 0.040$).

Although we did not find any group difference in transition energies, comparing control energies of individual nodes ($E_o(i)$, where i is a brain region) between groups gave us interesting results. In particular, for the transition from State 4 to State 1, right-parahippocampal cortex (R-PHC), right-putamen (R-Put) and left-pallidum (L-Pal) required higher energetic input in HCs than MDDs (R-PHC: t = 2.10, df = 51, p = 0.04, uncorrected independent t-test, R-Put: t = 4.04, df = 51, p = 0.0001, uncorrected independent t-test and L-Pal: t = 2.08, df = 51, p = 0.04, uncorrected independent t-test), whereas left-paracentral lobule (L-PC) required more control energy in MDDs than HCs (t = -2.85, p = 0.006, df = 51, uncorrected independent t-test) for the transition from State 4 to State 1. On the other hand, for the transition from State 3 to State 2, left-paracentral lobule (L-PC) required higher external control energy in HCs than MDDs (t =2.64, p =0.01, df = 51, uncorrected independent t-test) and left-transversetemporal cortex (L-TT) required higher control energy in MDDs than HCs (t = -2.25, p = 0.028, df = 51, uncorrected independent t-test). We show these group differences in control energies in Supplementary

Figure 5c as a violin plot and associated control signals in Fig 5a and 5c in the main text, respectively.

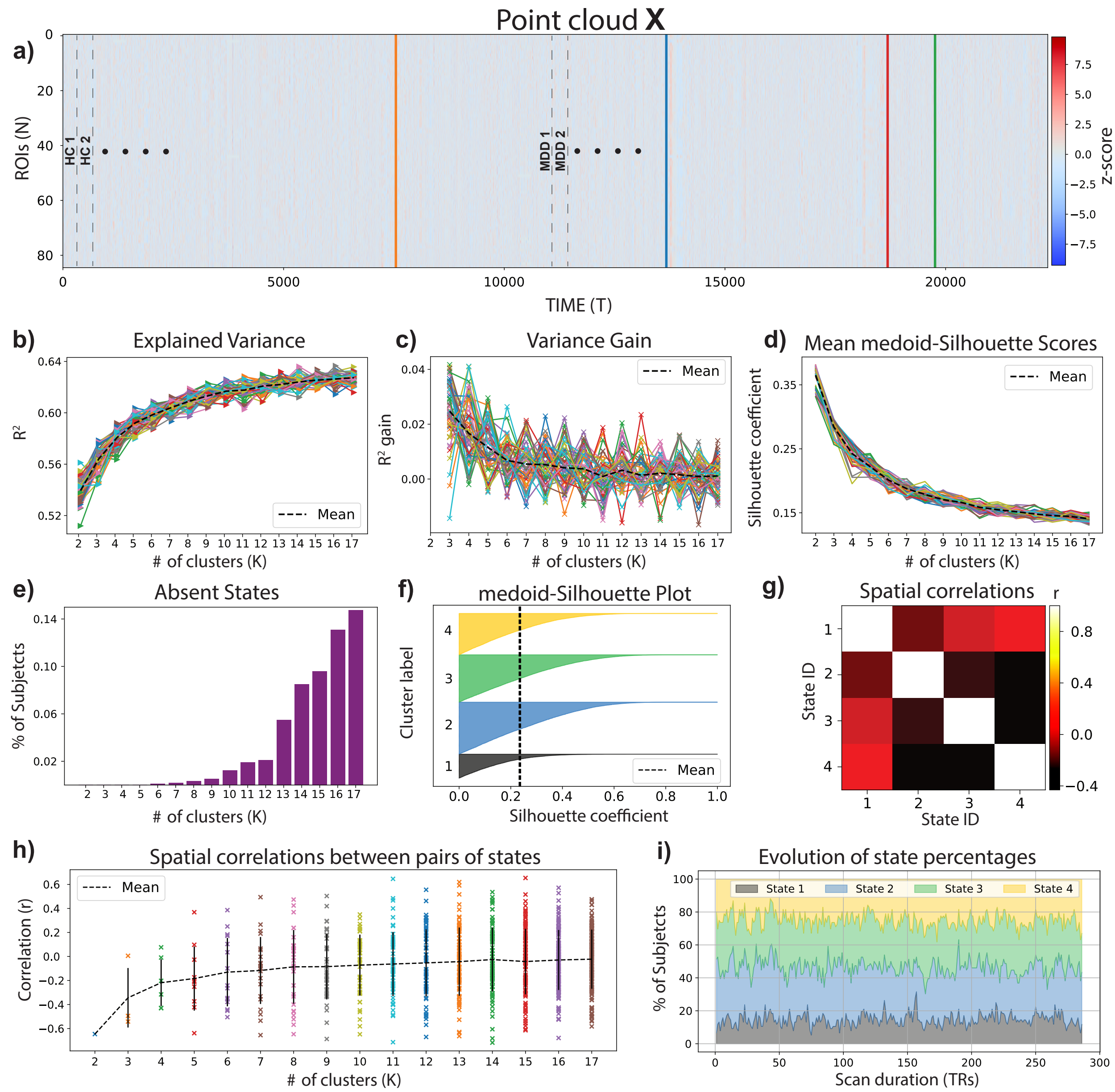

Supplementary Figure 1: Choice of optimal K**. a)** We show concatenated and z-scored time series $\mathbf{X}$. We highlight extracted cluster centers 1, 2, 3 and 4 in (blue, red, orange and green bars, respectively). Notice the time point representing cluster 3 belongs to an HC whereas clusters 1, 2 and 4 belong to MDD patients. We plot **b)** explained variance, **c)** gain in explained variance for unit increase in K, and **d)** mean medoid-silhouette score as a function of number of clusters K for 50 repetitions of k-medoids algorithm (colored lines) and the average values across repetitions (black dashed lines). **e)** We show a histogram of percentages of absent states across iterations and participants. **f)** We plot distributions of medoid-silhouette coefficients of each point in every cluster and highlight the mean silhouette coefficient with black dashed line. **g)** We calculate spatial similarity between every pair of extracted states. Observe that states are spatially non-correlated and non-overlapping. **h)** We plot the spatial correlation values between each pair of states as a function of K. Black dashed line shows the mean value for each K, and the error bars represent standard deviations from the mean. **i)** We plot the time evolution of percentages of subjects that belong to a given state during the duration of scans measured by repetition times (TR).

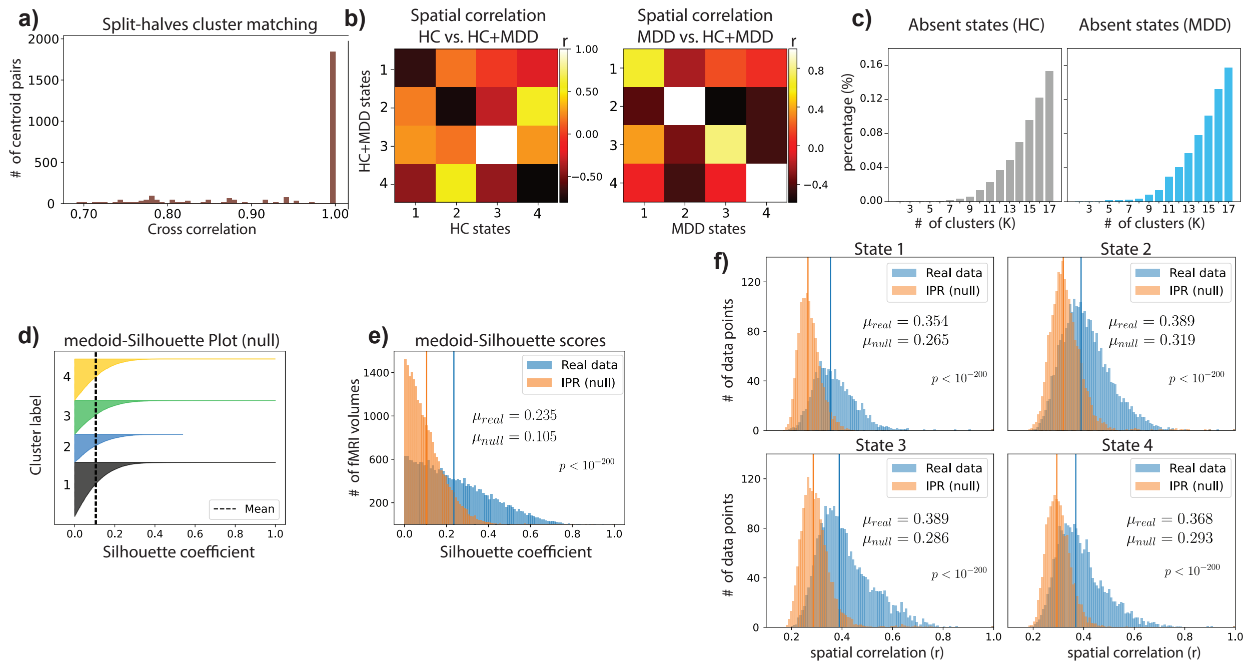

Supplementary Figure 2: Cluster Validation. a) We plot maximum cross-correlation values between each half of clusters obtained by split-half method. **b)** Heatmaps show spatial correlations between the clusters found in the main text and cluster found only in HCs (left) and only in MDDs (right). Clusters are matched so that the diagonal entries show the highest correlation. **c)** Absent states found across clinical populations when clusters are extracted separately from clinical groups. **d)** medoid-Silhouette plot for the IPR null distribution with maximum inertia. **e)** We also plot a histogram of silhouette coefficients comparing between the null and real data. Independent t-test shows there is a clear separation between two distributions. **f)** We show distributions of intra-cluster variance as the correlation distance between every pair of points within a given cluster and compare real data and IPR null model for each state $k = 1,2,3,4$. p values are calculated from independent t-tests.

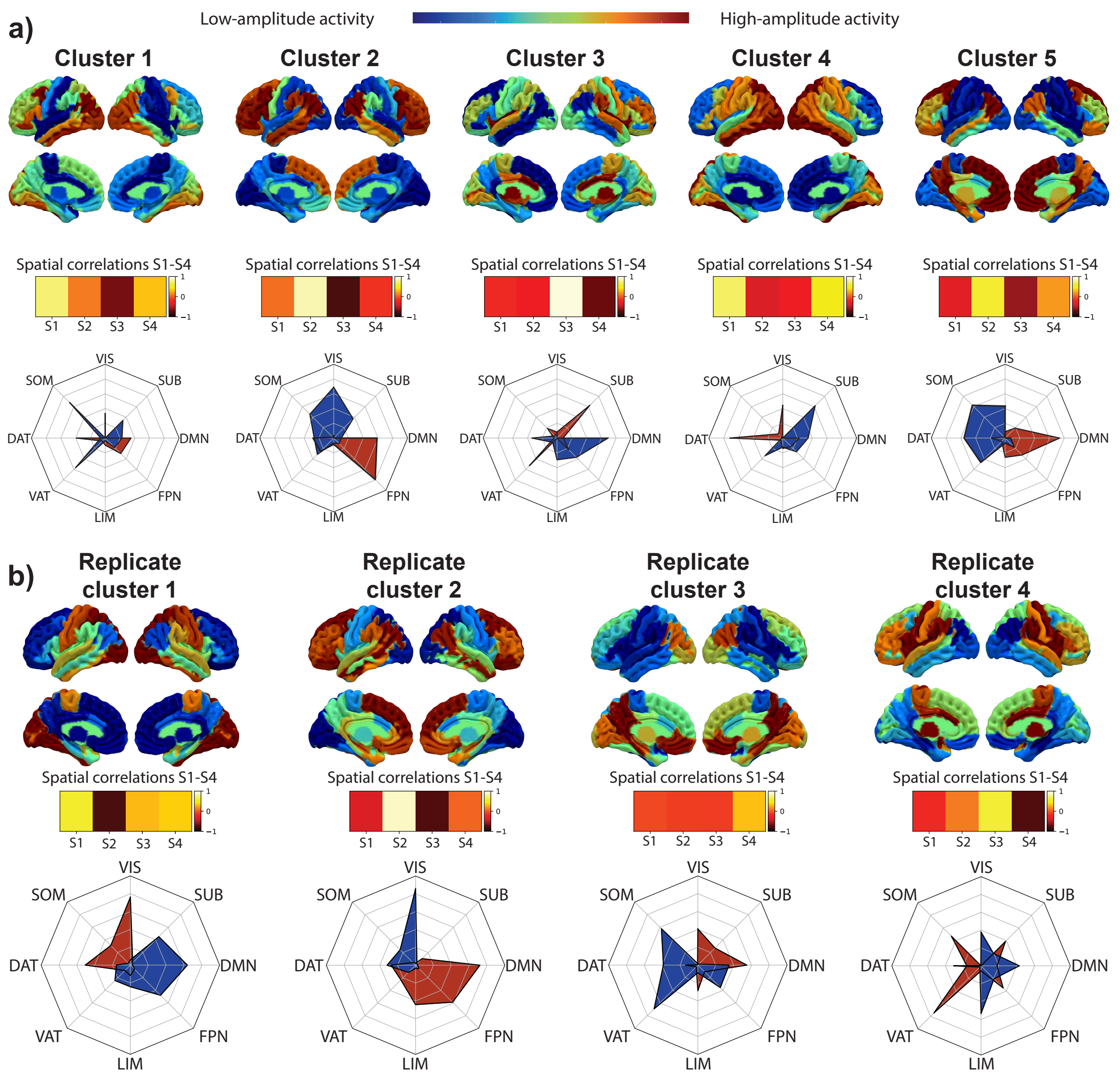

Supplementary Figure 3: Brain state robustness. a) We show cluster centroids as activation maps for $K = 5$. Below each map, we demonstrate degree of spatial similarity with original clusters studied in the main text (S1, S2, S3, S4) as a heatmap, and high and low amplitude components of the BOLD signal as canonical resting state networks. Observe that there is a high spatial similarity between these networks and the networks found when $K = 4$ in the main text. The additional network found is the high-litude Default Mode network and low-amplitude ventral attention, dorsal attention, somatomotor and visual networks (Cluster 5) which resembles State 2. **b)** We also show cluster robustness in an independent rs-fMRI dataset of healthy controls and MDD patients. Below each replicate brain state, we plot spatial similarity with the 4 states studied in the main text (S1, S2, S3, S4). There is a high similarity between the states found in the main text and the ones found in this dataset.

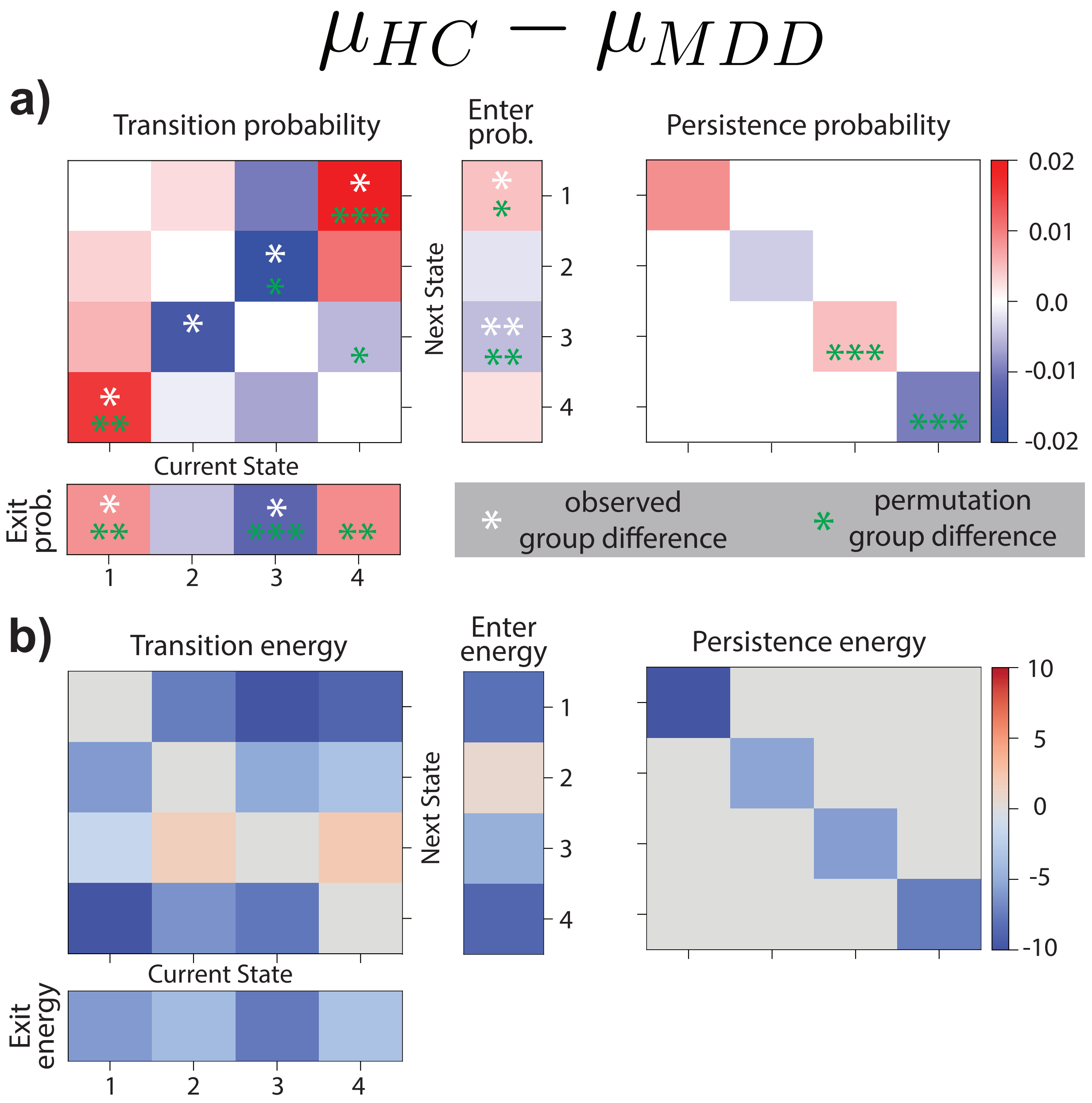

Supplementary Figure 4: Transition, persistence, exit and enter probabilities/energies. a) We show group mean difference (HC-MDD) of transition probabilities. Average of all rows in this matrix give exit probabilities whereas average of columns give enter probabilities. We show persistence probabilities separately on the right. *** $p < 0.001$, ** $p < 0.01$, * $p < 0.05$, black: independent t-test between clinical groups, green: independent t-test of permuted state sequences between clinical groups. **b)** Same with **a)** but instead for transition energies.

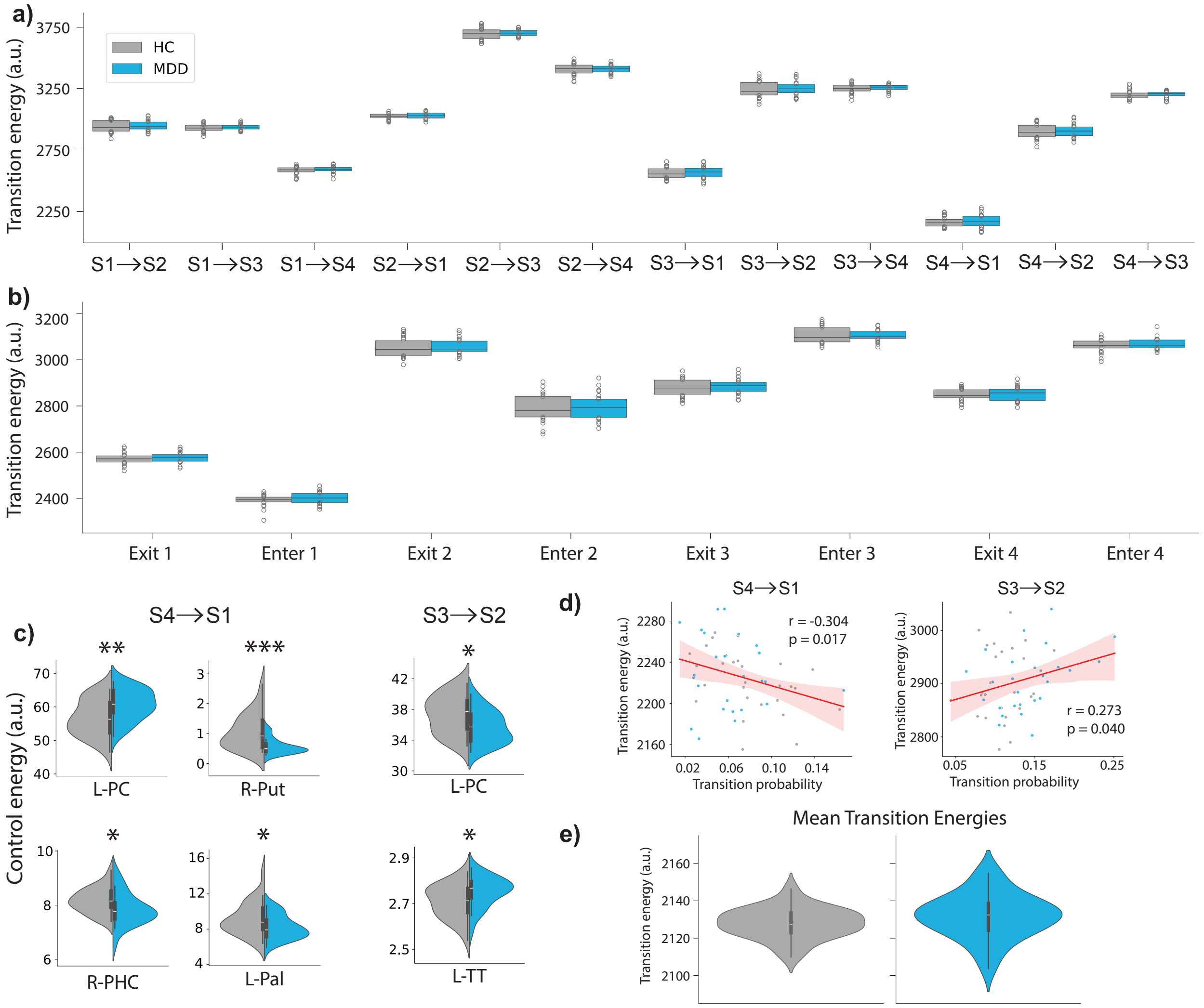

Supplementary Figure 5: Additional plots for network control analysis. a) We show transition energies between each pair of transitions split between clinical populations. **b)** Similarly, we show transition energies for exit and enter energies of each state. **c)** Violins show group difference in control energies for the network nodes whose control signals were plotted in Fig5a and d in the main text $^{*}p < 0.05$, $^{**}p < 0.01$ independent t-test. R-Put: Right Putamen, L-PC: Left paracentral lobule, R-PHC: Right parahippocampal gyrus, L-Pal: Left Pallidum and L-TT: Left transversetemporal cortex. **d)** We fit a single regression line for both groups' transition energies and transition probabilities for the transition from State 4 to State 1 (left) and from State 3 to State 2 (right). **e)** We show the trend in which mean transition energy averaged across all transitions (excluding persistence energies) are higher for MDD patients.

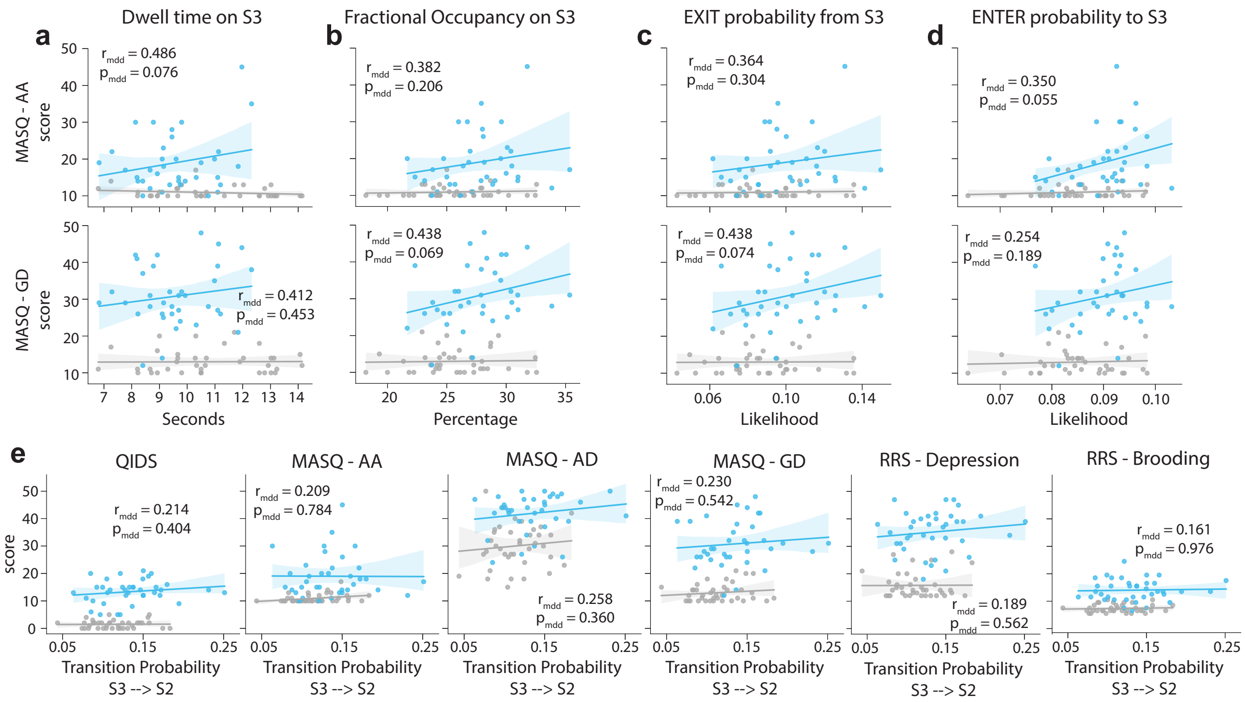

Supplementary Figure 6: Additional correlations with clinical scores. a) Correlations between dwell times on S3 and MASQ-Anxious Arousal (top) and MASQ-General Distress (bottom). **b)** Correlations between fractional occupancy on S3 and MASQ-Anxious Arousal (top) and MASQ-General Distress (bottom). **c)** Correlations between exit probability from State 3 and MASQ-Anxious Arousal (top) and MASQ-General Distress (bottom). **d)** Correlations between enter probability to State 3 and MASQ-Anxious Arousal (top) and MASQ-General Distress (bottom). Observe that although these correlations are not statistically significant, the trend is similar to the other two scores looked at in the main text (QIDS and MASQ-AD) are similar. **e)** Correlations between transition probabilities from State 3 to State 2 and QIDS, MASQ-Anxious Arousal, MASQ-Anhedonic Depression and MASQ-General Distress, RSS-Depression Related, RRS-Brooding (from left to right).

	fMRI			
	**MDD (n = 38)**	**HC (n = 38)**	**Statistic χ^2 / t (df)**	**p-value**
**Male (frequency, %)**	17, 44.73%	18, 47.36%	0.053	0.82
**Age, years (mean ± SD)**	28.10 ± 6.68	30.76 ± 8.64	1.50 (74)	0.14
**Medication use (frequency, %)**	17, 44.73%	-	-	-
**QIDS**	13.37 ± 4.19	1.47 ± 1.45	16.55 (74)	1.39E-26*
**MASQ-AA**	19 ± 7.62	10.92 ± 1.53	6.31 (72)	2.00E-8*
**MASQ-AD**	41.94 ± 6.89	30.03 ± 7.81	6.85 (72)	2.05E-9*
**MASQ-GD**	30.81 ± 8.13	13.00 ± 3.15	12.37 (72)	1.69E-19*
**RRS-DR**	35.21 ± 7.26	15.76 ± 4.58	13.76 (74)	4.29E-22*
**RRS-BR**	13.5 ± 3.84	6.84 ± 1.46	9.84 (74)	4.22E-15*
**RRS-RF**	12.07 ± 3.16	7.42 ± 2.36	7.16 (74)	4.81E-10*
	DTI			
	**MDD (n = 26)**	**HC (n = 27)**	**Statistic χ^2 / t (df)**	**p-value**
**Male (frequency, %)**	13, 50.00%	14, 51.85%	0.018	0.89
**Age, years (mean ± SD)**	28.42 ± 6.59	31.04 ± 8.00	1.27 (51)	0.21
**Medication use (frequency, %)**	12, 46.15%	-	-	-
**QIDS**	13.15 ± 4.10	1.37 ± 1.34	13.90 (51)	5.67E-19*
**MASQ-AA**	17.56 ± 5.58	10.96 ± 1.62	5.76 (50)	5.14E-7*
**MASQ-AD**	43.52 ± 6.31	30.15 ± 6.69	7.26 (50)	2.38E-9*
**MASQ-GD**	29.80 ± 7.87	13.04 ± 3.19	10.00 (50)	1.62E-13*
**RRS-DR**	33.19 ± 7.35	15.48 ± 3.97	10.74 (51)	1.04E-14*
**RRS-BR**	12.73 ± 3.97	6.88 ± 1.44	7.02 (51)	5.05E-9*
**RRS-RF**	11.57 ± 2.93	7.29 ± 2.35	5.75 (51)	4.98E-7*

Supplementary Table 1: Demographic and clinical characteristics. Quick Inventory of Depressive Symptomatology (QIDS), Mood and Anxiety Symptoms Questionnaire (MASQ) sub-scores: General Distress (GD), Anhedonic Depression (AD), and Anxious Arousal (AA), Ruminative Responses Scale (RRS) sub-scores: Depression-Related (DR), Brooding (BR), Reflection (RF).

ROI	State 1	State 2	State 3	State 4	LIM (%)	DMN (%)	FPN (%)	VAT (%)	SOM (%)	DAT (%)	VIS (%)	SUB (%)
**rh-lateralorbitofrontal**	Low	High	Low	Low	100	--	--	--	--	--	--	--
**rh-parsorbitalis**	Low	High	Low	Low	--	17.1	82.8	--	--	--	--	--
**rh-frontalpole**	Low	High	Low	High	100	--	--	--	--	--	--	--
**rh-medialorbitofrontal**	Low	High	Low	High	98.6	1.3	--	--	--	--	--	--
**rh-parstriangularis**	Low	High	Low	Low	--	1.3	36.8	61.8	--	--	--	--
**rh-parsopercularis**	Low	High	Low	Low	--	--	11.8	88.1	--	--	--	--
**rh-rostralmiddlefrontal**	Low	High	High	Low	--	--	100	--	--	--	--	--
**rh-superiorfrontal**	Low	High	Low	High	--	100	--	--	--	--	--	--
**rh-caudalmiddlefrontal**	Low	High	Low	Low	--	--	100	--	--	--	--	--
**rh-precentral**	Low	Low	High	Low	--	--	--	--	100	--	--	--
**rh-paracentral**	Low	Low	High	High	--	--	--	--	100	--	--	--
**rh-rostralanteriorcingulate**	Low	High	High	Low	--	98.6	1.3	--	--	--	--	--
**rh-caudalanteriorcingulate**	Low	High	High	Low	--	--	--	100	--	--	--	--
**rh-posteriorcingulate**	Low	High	High	Low	--	--	--	100	--	--	--	--
**rh-isthmuscingulate**	Low	High	Low	High	--	100	--	--	--	--	--	--
**rh-postcentral**	Low	Low	High	High	--	--	--	--	100	--	--	--
**rh-supramarginal**	Low	Low	High	Low	--	--	--	100	--	--	--	--
**rh-superiorparietal**	High	Low	High	High	--	--	--	--	--	100	--	--
**rh-inferiorparietal**	High	High	Low	Low	--	23.6	17.1	--	--	59.2	--	--
**rh-precuneus**	Low	Low	High	High	--	22.3	1.3	--	--	76.3	--	--
**rh-cuneus**	High	Low	High	High	--	--	--	--	--	--	100	--
**rh-pericalcarine**	High	Low	High	High	--	--	--	--	--	--	100	--
**rh-lateraloccipital**	High	Low	High	High	--	--	--	--	--	--	100	--
**rh-lingual**	High	Low	High	High	--	--	--	--	--	--	100	--
**rh-fusiform**	High	Low	High	High	1.3	--	--	--	--	--	98.6	--
**rh-parahippocal**	Low	Low	Low	High	25	--	--	--	--	--	75	--
**rh-entorhinal**	Low	Low	Low	High	98.6	--	--	--	--	--	1.3	--
**rh-temporalpole**	Low	Low	Low	High	96.0	1.3	--	2.6	--	--	--	--
**rh-inferiortemporal**	High	Low	Low	High	59.2	--	--	--	--	40.7	--	--
**rh-middletemporal**	Low	High	Low	High	1.3	96.0	--	--	--	2.6	--	--
**rh-bankssts**	Low	Low	Low	High	--	--	--	92.1	7.8	--	--	--
**rh-superiortemporal**	Low	Low	High	High	--	--	--	--	100	--	--	--
**rh-transversetemporal**	Low	Low	High	High	--	--	--	--	100	--	--	--
**rh-insula**	Low	Low	High	Low	--	--	--	100	--	--	--	--
**Right-Thalamus_Proper**	Low	Low	High	Low	--	--	--	--	--	--	--	100
**Right-Caudate**	Low	High	High	Low	--	--	--	--	--	--	--	100
**Right-Putamen**	Low	High	High	Low	--	--	--	--	--	--	--	100
**Right-Pallidum**	Low	High	High	Low	--	--	--	--	--	--	--	100
**Right-Accumbens_area**	Low	Low	High	Low	--	--	--	--	--	--	--	100
**Right-Hippocus**	Low	Low	Low	High	--	--	--	--	--	--	--	100
**Right-Amygdala**	Low	Low	Low	High	--	--	--	--	--	--	--	100
**Right-VentralDC**	Low	Low	High	Low	--	--	--	--	--	--	--	100
**lh-lateralorbitofrontal**	Low	High	Low	Low	100	--	--	--	--	--	--	--
**lh-parsorbitalis**	High	High	Low	High	--	100	--	--	--	--	--	--
**lh-frontalpole**	Low	High	Low	High	100	--	--	--	--	--	--	--
**lh-medialorbitofrontal**	Low	High	Low	High	100	--	--	--	--	--	--	--
**lh-parstriangularis**	High	High	Low	High	--	90.7	9.2	--	--	--	--	--
**lh-parsopercularis**	High	High	Low	Low	--	1.3	5.2	93.4	--	--	--	--
**lh-rostralmiddlefrontal**	High	High	Low	Low	--	--	100	--	--	--	--	--

**lh-superiorfrontal**	Low	High	Low	High	--	100	--	--	--	--	--	--
**lh-caudalmiddlefrontal**	High	High	Low	High	--	39.4	50	--	--	10.5	--	--
**lh-precentral**	High	Low	High	High	--	--	--	--	98.6	1.3	--	--
**lh-paracentral**	Low	Low	High	High	--	--	--	--	100	--	--	--
**lh-rostralanteriorcingulate**	Low	High	Low	High	--	100	--	--	--	--	--	--
**lh-caudalanteriorcingulate**	Low	Low	High	Low	--	--	--	100	--	--	--	--
**lh-posteriorcingulate**	Low	High	High	Low	--	--	--	100	--	--	--	--
**lh-isthmuscingulate**	Low	High	Low	High	--	100	--	--	--	--	--	--
**lh-postcentral**	Low	Low	High	High	--	--	--	--	100	--	--	--
**lh-supramarginal**	High	High	Low	Low	--	--	--	100	--	--	--	--
**lh-superiorparietal**	High	Low	High	High	--	--	--	--	--	100	--	--
**lh-inferiorparietal**	High	High	Low	High	--	96.0	--	--	--	2.6	1.3	--
**lh-precuneus**	High	Low	Low	High	--	92.1	--	--	--	7.8	--	--
**lh-cuneus**	High	Low	High	High	--	--	--	--	--	--	100	--
**lh-pericalcarine**	High	Low	High	High	--	--	--	--	--	--	100	--
**lh-lateraloccipital**	High	Low	High	High	--	--	--	--	--	--	100	--
**lh-lingual**	High	Low	High	High	--	--	--	--	--	--	100	--
**lh-fusiform**	High	Low	High	High	3.9	--	--	--	--	--	96.0	--
**lh-parahippocal**	Low	Low	Low	High	53.9	--	--	--	--	--	46.0	--
**lh-entorhinal**	Low	Low	Low	High	100	--	--	--	--	--	--	--
**lh-temporalpole**	Low	Low	Low	High	100	--	--	--	--	--	--	--
**lh-inferiortemporal**	High	Low	Low	High	71.0	1.3	--	--	--	27.6	--	--
**lh-middletemporal**	High	High	Low	High	--	100	--	--	--	--	--	--
**lh-bankssts**	High	High	Low	High	--	82.8	--	14.4	2.6	--	--	--
**lh-superiortemporal**	Low	Low	High	High	--	--	--	--	100	--	--	--
**lh-transversetemporal**	Low	Low	High	High	--	--	--	--	100	--	--	--
**lh-insula**	Low	Low	High	Low	--	--	--	100	--	--	--	--
**Left-Thalamus_Proper**	Low	Low	High	Low	--	--	--	--	--	--	--	100
**Left-Caudate**	Low	High	High	Low	--	--	--	--	--	--	--	100
**Left-Putamen**	Low	Low	High	Low	--	--	--	--	--	--	--	100
**Left-Pallidum**	High	High	High	Low	--	--	--	--	--	--	--	100
**Left-Accumbens_area**	Low	Low	High	Low	--	--	--	--	--	--	--	100
**Left-Hippocus**	Low	Low	Low	High	--	--	--	--	--	--	--	100
**Left-Amygdala**	Low	Low	Low	High	--	--	--	--	--	--	--	100
**Left-VentralDC**	Low	Low	High	Low	--	--	--	--	--	--	--	100
**brainstem**	Low	Low	High	Low	--	--	--	--	--	--	--	100

Supplementary Table 2: Characterization of regions of interests in the analysis. Column 1: Name of ROI from the Lausanne2018 parcellation. Columns 2-5: Component of activity (either high amplitude or low amplitude) on each ROI for State 1, State 2, State 3 and State 4 (from left to right). Columns 6-13: Belonging of each ROI to cognitive systems in terms of percentages of subjects computed using maximum dice score (n = 76). From left to right, LIM-Limbic network, DMN-Default mode network, FPN-Frontoparietal network, VAT-Ventral attention network, SOM-Somatomotor network, DAT-Dorsal attention network, VIS-Visual network, SUB-Subcortical network.

REFERENCES

1. Kaufman, L. & Rousseeuw, P. J. Clustering by means of medoids. *In: Dodge Y (ed) Statistical Data Analysis Based on the L 1 Norm and Related Methods* 405–416 (1987).

2. Mathieu, T., Yurchak, R., Birodkar, V. & Contributors. Scikit-learn-extra – a set of useful tools compatible with scikit-learn. Preprint at https://github.com/scikit-learn-contrib/scikit-learn-extra (2020).

3. Lenssen, L. & Schubert, E. Medoid Silhouette clustering with automatic cluster number selection. *Inf Syst* **120**, 102290 (2024).

4. Goutte, C., Toft, P., Rostrup, E., Nielsen, F. Å. & Hansen, L. K. On Clustering fMRI Time Series. *Neuroimage* **9**, 298–310 (1999).

5. Gutierrez-Barragan, D., Basson, M. A., Panzeri, S. & Gozzi, A. Infraslow state fluctuations govern spontaneous fMRI network dynamics. *Current Biology* **29**, 2295–2306 (2019).

6. Cornblath, E. J., Ashourvan, A. & Kim, J. Z. et al. Temporal sequences of brain activity at rest are constrained by white matter structure and modulated by cognitive demands. *Commun Biol* **3**, (2020).

7. Liégeois, R., Laumann, T. O., Snyder, A. Z., Zhou, J. & Yeo, B. T. T. Interpreting temporal fluctuations in resting-state functional connectivity MRI. *Neuroimage* **163**, 437–455 (2017).

8. Jacob, Y. *et al.* Neural correlates of rumination in major depressive disorder: A brain network analysis. *Neuroimage Clin* **25**, 102142 (2020).

9. Parkes, L., Kim, J. Z. & Stiso, J. et al. A network control theory pipeline for studying the dynamics of the structural connectome. *Nat Protoc* **19**, (2024).
